# Genetic analysis and QTL mapping of domestication-related traits in chili pepper (*Capsicum annuum* L*.*)

**DOI:** 10.3389/fgene.2023.1101401

**Published:** 2023-05-15

**Authors:** Hector Lopez-Moreno, Ana Celia Basurto-Garduño, Maria Alejandra Torres-Meraz, Eric Diaz-Valenzuela, Sergio Arellano-Arciniega, Juan Zalapa, Ruairidh J. H. Sawers, Angelica Cibrián-Jaramillo, Luis Diaz-Garcia

**Affiliations:** ^1^ Ecological and Evolutionary Genomics Laboratory, Unidad de Genomica Avanzada (Langebio), Irapuato, Mexico; ^2^ Instituto Nacional de Investigaciones Forestales, Agricolas y Pecuarias Campo Experimental AGS, Pabellón de Arteaga, Mexico; ^3^ Department of Horticulture, University of WI-Madison, Madison, WI, United States; ^4^ USDA-ARS Vegetable Crops Research Unit, Department of Horticulture University of WI-Madison, Madison, WI, United States; ^5^ Department of Plant Science, The Pennsylvania State University, State College, PA, United States

**Keywords:** *Capsicum*, domestication, domestication syndrome, chiltepin, reciprocal translocation, linkage map, QTL mapping, wild relative

## Abstract

Chili pepper (*Capsicum annuum* L.) is one of the oldest and most phenotypically diverse pre-Columbian crops of the Americas. Despite the abundance of genetic resources, the use of wild germplasm and landraces in chili pepper breeding is limited. A better understanding of the evolutionary history in chili peppers, particularly in the context of traits of agronomic interest, can contribute to future improvement and conservation of genetic resources. In this study, an F_2_:_3_ mapping population derived from a cross between a *C. annuum* wild accession (Chiltepin) and a cultivated variety (Puya) was used to identify genomic regions associated with 19 domestication and agronomic traits. A genetic map was constructed consisting of 1023 single nucleotide polymorphism (SNP) markers clustered into 12 linkage groups and spanning a total of 1,263.87 cM. A reciprocal translocation that differentiates the domesticated genome from its wild ancestor and other related species was identified between chromosomes 1 and 8. Quantitative trait locus (QTL) analysis detected 20 marker-trait associations for 13 phenotypes, from which 14 corresponded to previously identified loci, and six were novel genomic regions related to previously unexplored domestication-syndrome traits, including form of unripe fruit, seedlessness, deciduous fruit, and growth habit. Our results revealed that the genetic architecture of *Capsicum* domestication is similar to other domesticated species with few loci with large effects, the presence of QTLs clusters in different genomic regions, and the predominance of domesticated recessive alleles. Our analysis indicates the domestication process in chili pepper has also had an effect on traits not directly related to the domestication syndrome. The information obtained in this study provides a more complete understanding of the genetic basis of *Capsicum* domestication that can potentially guide strategies for the exploitation of wild alleles.

## 1 Introduction

Chili pepper (*Capsicum* sp.) is one of the oldest domesticated crops in the Americas (Davenport, 1970). Currently, chili pepper production exceeds 60 million tons annually (FAOSTAT, 2020), with cultivation in more than 140 countries and an annual revenue of 50 billion dollars. The *Capsicum* genus includes more than 30 species, from which only *C. annuum, C. frutescens*, *C. chinense*, *C. baccatum* and *C. pubescens* are considered domesticated. *C. annuum* is the most phenotypically diverse and broadly distributed species in the genus (Tripodi and Kumar, 2019). Genetic and anthropological evidence suggest *C. annuum* was domesticated from the wild chiltepin pepper (*Capsicum annuum* var*. glabriusculum*) about 8,000 years ago in a region spanning tropical and subtropical America (Davenport, 1970; Hunziker, 2001; Perry et al., 2007). The major differences between cultivated and wild chili peppers are related to fruit morphological and metabolomic traits (Kumar et al., 2018). The chiltepin parent has small, round fruits which are erect, highly pungent, and dehiscent, and chiltepin fruit are primarily eaten by birds and picked from wild stands by humans (Tewksbury and Nabhan, 2001; Kumar et al., 2018). In contrast, cultivated peppers vary in their degree of pungency and external appearance, although they are typically large, elongated, pendant and non-dehiscent, remaining on the plant until harvest. Additionally, domesticated and wild accessions of *Capsicum* differ in other traits that could play an important role in their fitness and local adaptation such as plant architecture, phenology and physiology. Typically, chiltepin plants are highly branched perennial shrubs with small, round leaves that can become climbing plants (Hernandez-Verdugo et al., 1999; Hayano-Kanashiro et al., 2016). In the wild, these plants can be found in arid regions and their immature stems and fruits produce pigments as protection against UV radiation (Borovsky et al., 2004). On the other hand, due to their adaptation to agricultural production systems, specially those in greenhouse conditions, domesticated chili plants are compact, annual in habit, with large leaves and have little production of pigments, with likely an array of additional adaptations to the conditions of greenhouses and monocultures.

Previous genetic mapping studies of crop domestication traits have identified genomic regions that govern important differences between wild and cultivated plants (Paterson et al., 1988; Doebley et al., 1995; Li et al., 2006; Simons et al., 2006; Dempewolf et al., 2012). Based on the results of these studies, Ross-Ibarra (2005) proposed the generalization that crop domestication is driven by a low number of loci with relatively large effects and a preponderance of recessive action. Although these studies have focused on the major traits associated with the species domestication syndrome, the consequences of domestication, ongoing improvement and dispersal to new environments have been reported to have broader impacts on cultivated plants (Hernández-Terán et al., 2017).

In the current study, a quantitative trait locus (QTL) mapping analysis was conducted in an F_2_:_3_ population derived from Puya, a domesticated accession, and Chiltepin, a wild ancestor of cultivated pepper, to identify genomic regions associated with traits of agronomic importance or related to the domestication syndrome. We identified novel marker-trait associations by combining standard field evaluation and computer vision strategies using 19 phenotypic traits related with fruit and leaf morphology, phenology and plant architecture. In addition, our QTL analysis showed that most of the traits analyzed share patterns such as few loci of relatively large effect, preponderance of domesticated recessive loci, clustering of QTLs in certain genomic regions or pleiotropic loci, and biased effect of QTLs toward the domesticated phenotype for strongly selected traits.

## 2 Materials and methods

### 2.1 Plant material and growth conditions

**FIGURE 1:**
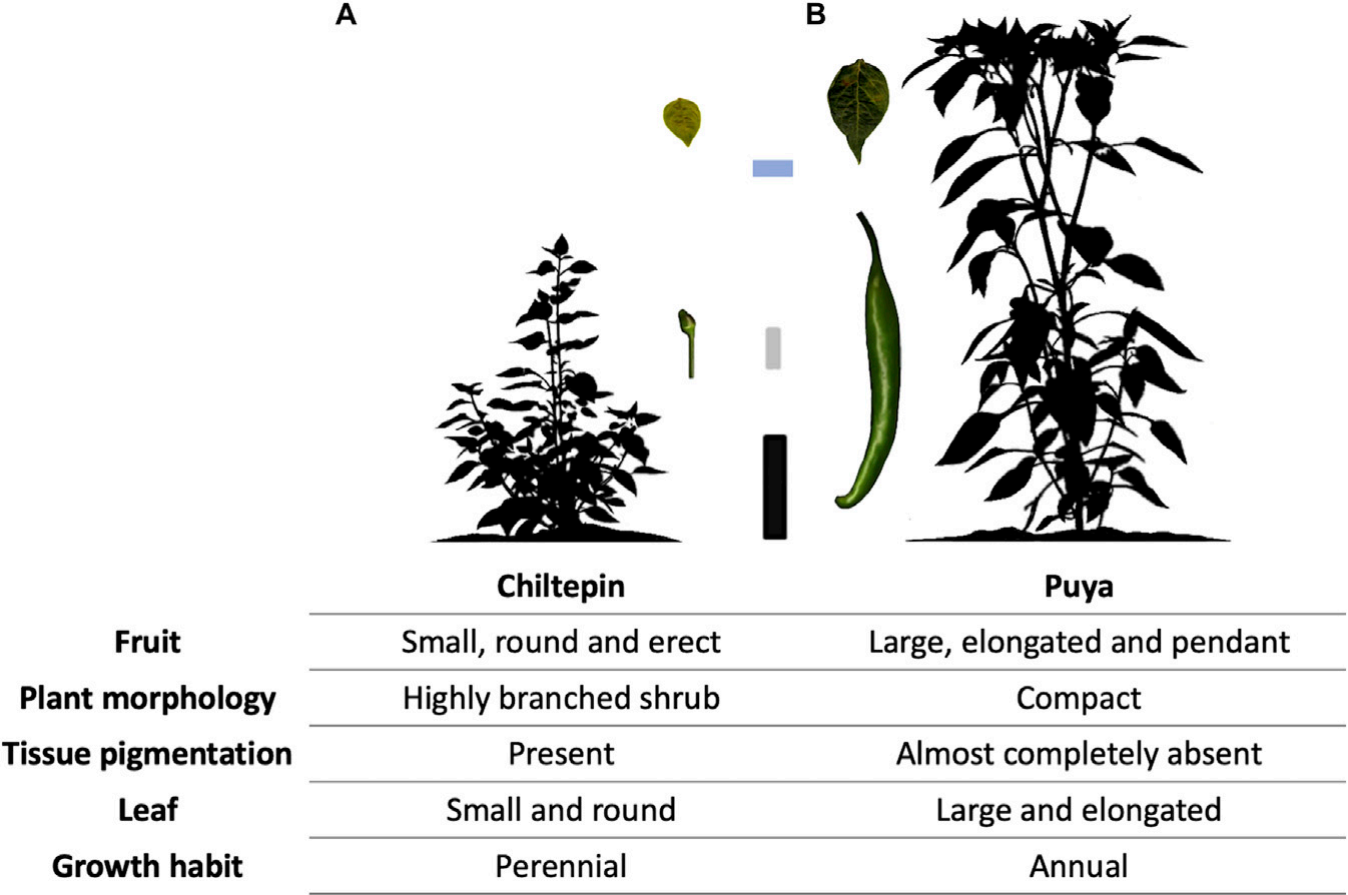
Fruit and plant characteristics of the wild (Chiltepin) and domesticated (Puya) chili pepper parental accessions used to study agronomic and domestication-related traits in an F_2_:_3_ Puya × Chiltepin population. **(A)** Image of plant and fruit of Chiltepin. **(B)** Image of plant and fruit of Puya. 10 cm black scale bar for plants at 70 days after sowing. 1 cm gray scale bar for the fruits. 2 cm blue scale bar for the leaves.

A biparental chili pepper mapping population of 153 F_2_:_3_ families was derived from a single F_1_ progeny generated from a cross between a wild accession (Chiltepin) and a domesticated accession (Puya) previously reported by Díaz-Valenzuela et al., 2020 (Figure 1). Dried fruits of the wild accession were collected from a wild population in Querétaro, México (Latitude 20.79°N and Longitude 99.87°W), and the fruits of the domesticated accession were obtained in Guanajuato, México (Latitude 20.67°N and Longitude 101.35°W). In order to carry out a replicated experiment, 153 F_2_ plants were grown in isolation in a controlled environment greenhouse and eventually self-pollinated to produce F_3_ families. F_3_ family seeds were germinated under greenhouse conditions following standard procedures as in Díaz-Valenzuela et al. (2020). Briefly, seeds were disinfected using a 10% bleach solution for 10 min at room temperature, scarified with 0.05 N HCl for 30 min and rinsed for 1 hour with distilled water. The seeds were sown at a depth of less than 10 mm in germination trays containing a mixture of peat moss, vermiculite, and perlite, in 3:1:1 proportion. Trays were maintained in darkness at about 70% humidity and at 25°C for 4 days and later moved to 24°C–27°C. After 2 months, the seedlings were transplanted to the field using a randomized complete block design (RCBD) with three replicates and eight plants per plot. The experiment was conducted in a high tunnel and plastic mulch in Aguascalientes, Mexico (Latitude 22°4′N and Longitude 102°16′W, elevation 1894 masl).

### 2.2 Phenotypic evaluation

A total of 19 agronomic and domestication-related traits were evaluated according to previous studies (Han et al., 2016; Chunthawodtiporn et al., 2018) with some modifications (Table 1). All phenotypes were evaluated on a RCBD replicated (3x8) trial of the 153 F_3_ families, which are replicated plants derived from self-pollinated 153 F_2_ individuals. As described in Table 1, some phenotypes were also measured on the individual F_2_ parents. To evaluate *deciduous fruit*, manual assessments of the force required to remove the peduncle from the fruit (previously separated from the plant) were conducted by one person. *Fruit morphology* was measured using image analysis (on a flat surface scanner) based on five fruits randomly harvested from the eight plants of each experimental unit. The images were further processed as in Diaz-Garcia et al., 2018 with the R packages EBImage (Pau et al., 2010) and Momocs (Bonhomme et al., 2014). *Growth habit* was determined by assessing the phenological stage of the plants in each replicate 120 days after transplanting. *Perenniality* was evaluated by assessing senescence at 120 days; specifically, dead or senescent plants were considered annuals, whereas plants that remained green were considered perennials.

**TABLE 1:** Summary of the chili pepper agronomic and domestication-related traits evaluated in an F_2_:_3_ Puya × Chiltepin population.

Phenotype	Type of variable	Method	Description	Stage	Numbers of observations per plot
Plant architecture					
Stem pigmentation (SP)^a^	Qualitative	Evaluated in the field	Green, Purple	60 DAT^c^	Average observation of 8 plants
Plant height (PH)	Quantitative	Evaluated in the field	From soil to head of the plant (cm)	80 DAT	Measurement of 3 plants
Main stem length (MSL)	Quantitative	Evaluated in the field	From soil to the first branch	80 DAT	Measurement of 3 plants
Branch angle (BA)	Qualitative	Evaluated in the field	Narrow (∼45°), Wide (∼90°)	80 DAT	Average observation of 8 plants
Fruit					
Unripe fruit pigmentation (UFP)^a^	Qualitative	Evaluated in the field	Green, Purple	60 DAT	Average observation of 8 plants
Fruit orientation (FO)^a^	Qualitative	Evaluated in the field	Pendant, Erect	90 DAT	Average observation of 8 plants
Form of unripe fruit (FUF)	Qualitative	Evaluated in harvested fruit	Wrinkled, Smooth	100 DAT	Average observation of 8 plants
Seedless fruit (SF)^b^	Qualitative	Evaluated in harvested fruit	Absence of seed, Presence of seed	Maturity 2018,2019	Average observation of 20 fruits
Deciduous fruit (DF)	Qualitative	Evaluated in harvested fruit	Non-deciduous, Deciduous	60 DAA^d^	Average observation of 20 fruits
Fruit weight (FWE)	Quantitative	Evaluated in harvested fruit	Weight of 10 fresh fruit	50 DAA	Total weight of 10 fresh fruits
Length (FL)	Quantitative	Evaluated in harvested fruit using image analysis	cm	50 DAA	5 fruits per image
Width (FWI)					
Area (FA)			cm^2^		
Shape (FS)			FL/FWI		
Leaf					
Length (LL)	Quantitative	Evaluated in harvested leaves using image analysis	cm	100 DAT	6 leaves per image
Width (LW)			cm		
Area (LA)			cm^2^		
Shape (LS)			LL/LW		
Phenology					
Growth habit (GH)	Qualitative	Evaluated in the field	Perenne, Annual	120 DAT	Average observation of 8 plants

^a^ Evaluated in populations F_2_ and F_3_.

^b^Only evaluated in the F_2_ individual plants.

^c^DAT- days after transplanting.

^d^DAA- days after anthesis.

### 2.3 Statistical analysis

Basic descriptive statistics (maximum, minimum, mean and standard deviation) and overall distribution were determined for the 11 non-binary traits. For the binary traits, 3:1 segregation patterns (consistent with a single major dominant gene) were tested based on a chi-square test for goodness-of-fit.

All phenotypic traits were analyzed following a mixed linear model in the R package lme4 (Bates et al., 2015). The mixed model was of the form =μ+Gi+Bj+εij , where yij was the phenotypic value of line i in block j ; μ denotes the general mean of the population; Gi was the effect of genotype; Bj was the effect of the block; and εij represents the error. Best Linear Unbiased Predictors (BLUPs) were used for QTL mapping. Genomic heritabilities were calculated in sommer (Covarrubias-Pazaran, 2016) following Covarrubias-Pazaran et al., 2018. The additive relationship matrix A for the heritability computations was constructed with the function A. mat from the rrBLUP package (Endelman, 2011).

### 2.4 Genotypic analysis and linkage map construction

Total genomic DNA was extracted from leaf tissue of the parental lines and the 153 F_2_ plants using Plant DNeasy mini kit (QIAGEN^®^) following the manufacturer’s instructions. DNA was quantified using a Qubit^®^ fluorometer based on 260 nm absorbance. DNA samples were analyzed using genotyping-by-sequencing (tGBS^®^) to discover single nucleotide polymorphism (SNP) markers. Reads were aligned to the *Capsicum* CM334 reference genome (Kim et al., 2014) by Data2bio^®^ for SNP calling. Variant calling format data was converted to ABH format and filtered by removing markers with more than 40% missing data in TASSEL software ver. 5.0 (Bradbury et al., 2007). Genotype-Corrector (Miao et al., 2018) was used for genotype correction and imputation considering a sliding window size of 15 markers and an error rate of 0.03. Markers with segregation distortion (*p* < 0.01, chi-square test) were discarded. Additionally, an imputation of missing data and correction of homozygous and heterozygous haplotypes was performed with ABHGenotypeR (Furuta et al., 2017), with a sliding window size of three markers. Finally, data were inspected visually to identify misordered markers in particular regions of the linkage map. The linkage map was constructed in ASMap (Taylor and Butler, 2017) using the Kosambi mapping function. Spearman correlation between linkage and physical (Kim et al., 2014) maps was used to assess the quality of the genetic linkage map.

### 2.5 QTL mapping analysis

For all the traits, QTL mapping was performed using the Haley-Knott regression method with the stepwise function from the R/qtl package (Broman et al., 2003). For each trait, the LOD threshold was determined based on a 1,000-permutation test at *α* = 0.05. Once the QTLs were determined, 1.8 LOD-supporting intervals were calculated with the lodint function. Additive and dominance effects and phenotypic variances for each QTL model were estimated using the fitqtl function.

## 3 Results

### 3.1 Phenotypic variation at the whole plant level showed predominance of the wild phenotype for traits directly and not-directly related to the domestication syndrome

**FIGURE 2:**
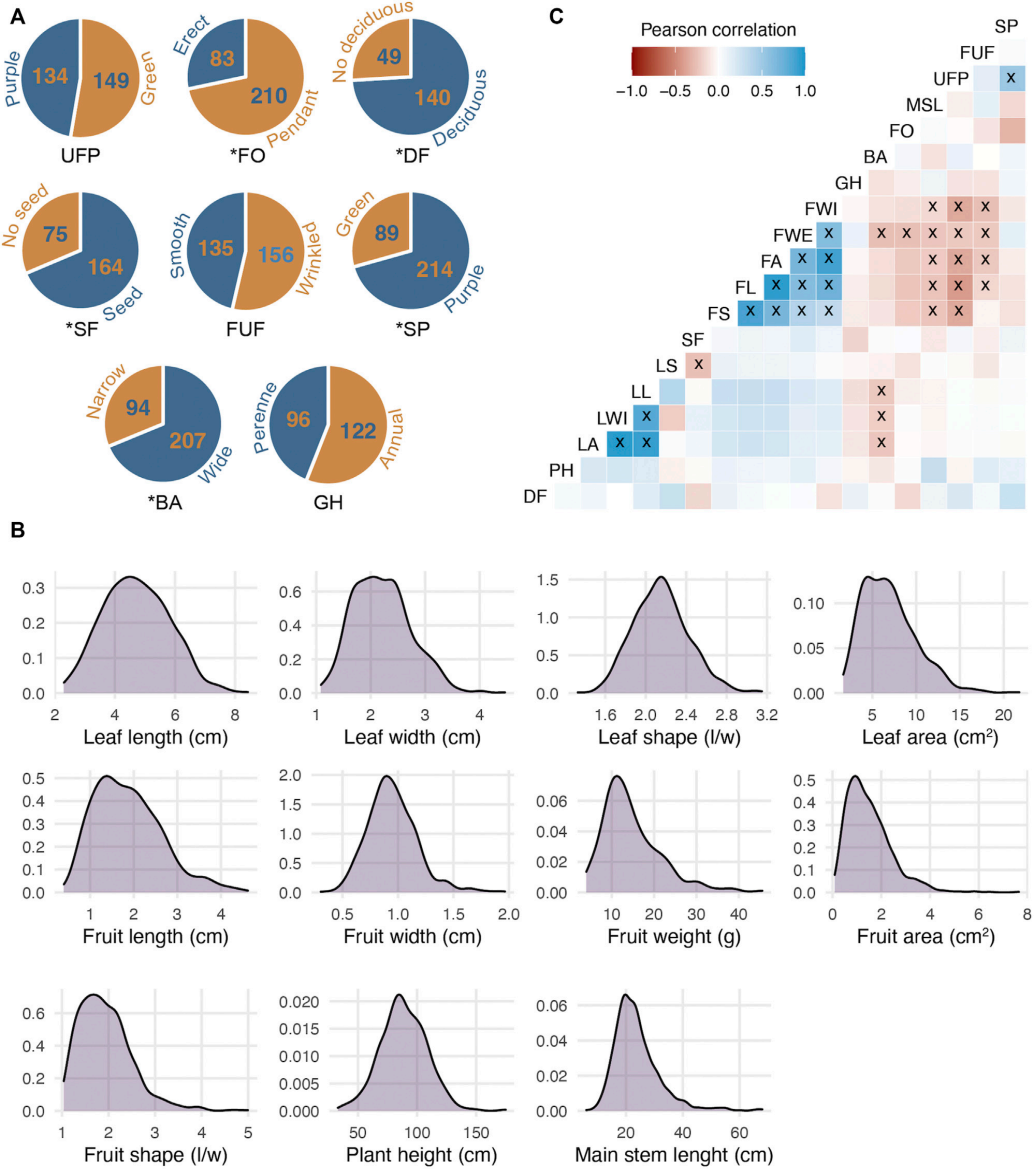
Phenotypic variation in a chili pepper F_2_:_3_ mapping population derived from a Puya x Chiltepin cross used to evaluate domestication-related traits. **(A)** Phenotypic ratios of eight qualitative traits; the wild phenotype contribution is shown in blue and the domesticated in orange (except for SF); * indicates that occurrences of each phenotype are in concordance with 1:3 ratio. **(B)** Frequency distribution of 11 quantitative traits. **(C)** Pearson’s correlation among 19 phenotypic traits; x indicates significant correlations (at *p* = 0.05). SP, stem pigmentation; FUF, form of unripe fruit; UFP, unripe fruit pigmentation; MSL, main stem length; FO, fruit orientation; BA, branch angle; GH, growth habit; FWI, fruit width; FWE, fruit weight; FA, fruit area; FL, fruit length; FS, fruit shape; SF, seedless fruit; LS, leaf shape; LL, leaf length; LWI, leaf width; LA, leaf area; PH, plant height; DF, deciduous fruit.

To analyze the phenotypic and genetic consequences of *Capsicum* domestication, a phenotypically highly contrasting cross between wild (‘Chiltepin’) and domesticated (‘Puya’) parents was used. The maternal parent ‘Puya’ is a landrace with highly pronounced domestication syndrome characteristics such as large-elongate, pendant and non-deciduous fruit, non-pigmented tissues and annual growth habit (Kumar et al., 2018). Conversely, Chiltepin possesses classic crop wild relative characteristics such as small-round, erect and deciduous fruit, pigmented tissues and perennial habit. In the wild, Chiltepin can take on a climbing habit and can reach a height of up to 2 m although its growth is slower compared to domesticated accessions such as Puya (Figure 1) (Nabhan, 1986; Hayano-Kanashiro et al., 2016). Variation for all phenotypic traits was observed in our F_2:3_ population (Table 2). For example, quantitative traits related with plant architecture such as *plant height* and *main stem length* varied between 34 and 174 cm, and 6 and 68 cm, respectively (Figure 2B). Qualitative traits such as *stem pigmentation* and *branch angle* showed a 3:1 ratio (*p*-value >0.05, *X* ^*2*^ test) in which purple stem and wide branch were the dominant phenotypes (Figure 2A). *Fruit size and shape* were highly correlated (average *r* ^2^ = 0.67, Figure 2C), and showed large phenotypic variation. For example, *fruit weight* ranged between 4.00 and 45.56 g per fruit, whereas *fruit area* ranged between 0.09 and 7.64 cm^2^, respectively (>1 order of magnitude in both cases). A 3:1 segregation pattern consistent with a single dominant gene was observed for *seedless fruit*, *deciduous fruit* and *fruit orientation* (*p*-value >0.05, *X* ^*2*^ test), in which seeded, deciduous and pendant were the dominant phenotypes. The *form of unripe fruit* and *unripe fruit pigmentation* showed ratios more similar to 1:1 (*p*-value > 0.05, *X* ^*2*^ test). Leaf size traits showed high correlation (average *r* ^2^ = 0.80) as well as variation. For example, *leaf area*, which was estimated digitally, showed variation of one order of magnitude (1.67–21.78 cm^2^). Great variation was also observed for *leaf shape* (1.32–3.14). Finally, a similar number of accessions showed perennial (96) and annual (122) growth habit characteristics, consistent with a 1:1 ratio (*p*-value >0.05, *X* ^*2*^ test) indicating that this trait is not governed by a single major gene.

**TABLE 2:** Basic descriptive statistics and genomic heritabilities for 19 phenotypic chili pepper agronomic and domestication-related traits used to study an F_2:3_ Puya × Chiltepin population.

Trait	Min	Max	Mean	SD	H^2^ G
Stem pigmentation	0	1	-	-	0.54
Plant height (cm)	33.00	175.00	88.69	19.61	0.39
Main stem length (cm)	6.00	68.00	23.80	8.16	0.19
Branch angle	0	1	-	-	0.07
Unripe fruit pigmentation	0	1	-	-	0.39
Fruit orientation	0	1	-	-	0.37
Form of unripe fruit	0	1	-	-	0.23
Seedless fruit	0	1	-	-	0.23
Deciduous fruit	0	1	-	-	0.49
Fruit weight (g)	4.00	45.56	14.71	6.99	0.55
Fruit length (cm)	0.39	4.61	1.87	0.77	0.61
Fruit width (cm)	0.30	1.96	0.94	0.22	0.43
Fruit area (cm2)	0.09	7.64	1.49	0.92	0.59
Fruit shape (FL/FWI)	1.03	4.99	1.93	0.56	0.51
Leaf length (cm)	2.30	8.42	4.71	1.09	0.46
Leaf width (cm)	1.08	4.46	2.21	0.52	0.21
Leaf area (cm2)	1.67	21.78	6.93	3.10	0.29
Leaf shape (LL/LW)	1.32	3.14	2.15	0.24	0.42
Growth habit	0	1	-	-	0.35

As mentioned above, five traits showed a 3:1 ratio (*p*-value >0.05, *X* ^*2*^ test). Three were dominant for the wild phenotype (deciduous fruit, pigmented stem and wide branch) and only pendant fruit was dominant for the cultivated one, seedless fruits could not be attributed to any parental phenotype (Figure 2A). For quantitative traits, Figure 2B shows that for most traits the distribution was skewed towards the wild-type phenotype. In general, the wild phenotype showed at least partial dominance for both domestication target and non-target traits, similar to previous observations in *Capsicum* (Diaz-Valenzuela et al., 2020). Genomic heritability estimates varied considerably among the phenotypes (Table 2). *Stem pigmentation* and most *fruit size and shape* traits showed medium to high heritability (>0.5), whereas traits related to *plant architecture*, *growth habit*, *leaf*, and all other *fruit-related* traits showed medium to low heritability (<0.5).

### 3.2 The genetic map confirmed a major translocation that differentiates domesticated peppers from the wild ancestor *Capsicum annuum* var*. glabriusculum*

In total, 1,023 polymorphic, high-quality SNP markers were used to build the linkage map. All markers were clustered into 12 linkage groups, consistent with the *Capsicum* chromosome haploid number (Mimura et al., 2012). The genetic map spanned 1,263.8 cM and linkage group sizes ranged from 75.3 (LG 8) to 174.2 cM (LG 1, Table 3). The mean gap distance was 1.2 cM, while the largest gap between markers was 10.7 cM.

**TABLE 3:** Description of a chili pepper linkage map constructed based on 1023 SNP markers and using an F_2__:__3_ Puya × Chiltepin mapping (n = 153) population.

LG	Number of SNPs	Total distance (cM)	Average distance between markers (cM)	Max gap between markers (cM)	Spearman´s correlation coefficient
1	124	174.22	1.40	9.13	0.99
2	113	114.3	1.01	8.19	0.99
3	121	136.5	1.12	6.04	0.99
4	60	78.21	1.3	10.2	1
5	88	87.63	0.99	4.92	0.99
6	81	95.69	1.18	10.76	1
7	63	89.91	1.42	6.38	1
8	42	75.39	1.79	6.44	0.99
9	90	116.21	1.29	7.26	0.95
10	69	94.82	1.37	9.32	1
11	76	98.37	1.29	9.59	0.99
12	97	102.62	1.05	6.24	0.99
Total/average	1,023*	1,263.87*	1.26**	7.87**	0.99**

* Total. **Average.

**FIGURE 3:**
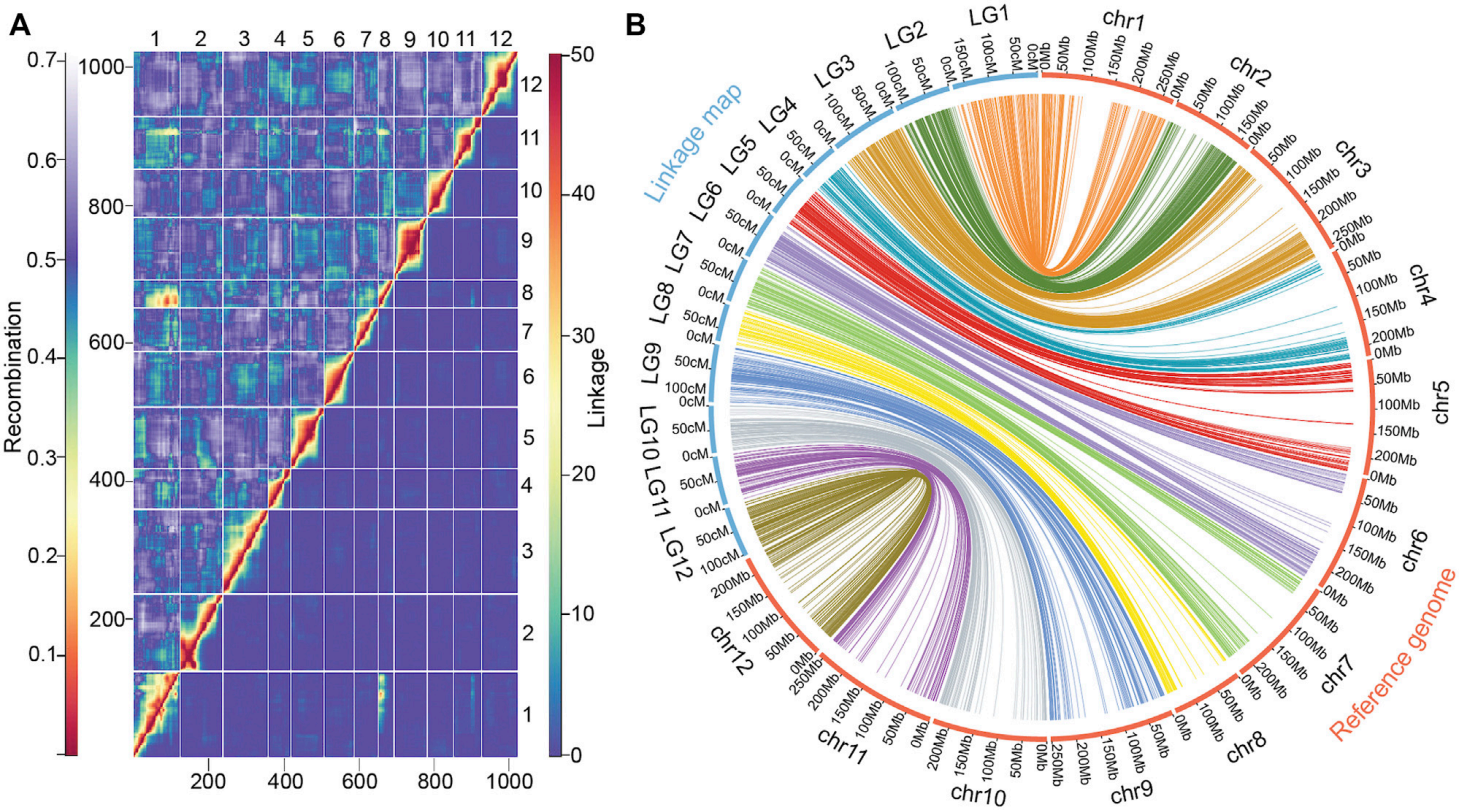
Recombination and linkage map collinearity of a chili pepper map (n = 153, F_2_:_3_ Puya × Chiltepin) with the physical genome. **(A)** Recombination fractions (upper triangle) and linkage LOD score (lower triangle) of the 1023 SNP markers used. **(B)** Collinearity between the linkage map and its reference genome (*Capsicum* CM334).

Pairwise recombinations and LOD scores, reflecting the strength of linkage between markers, were consistent for all linkage groups, indicating good quality of the genetic map. The linkage analysis detected a reciprocal translocation between chromosomes 1 and 8 (Figure 3A), previously characterized by Wu et al., 2009 and Park et al., 2014. The collinearity of each linkage group with the *Capsicum* reference genome was also inspected (Figure 3B); Spearman’s correlation coefficients were very high for all linkage groups, and varied between 0.95 and 1, with a mean value of 0.99 (Table 3).

### 3.3 Genetics of *Capsicum* domestication follows the pattern of a few large effect loci and a predominance of recessive alleles in the domesticated accession

**FIGURE 4:**
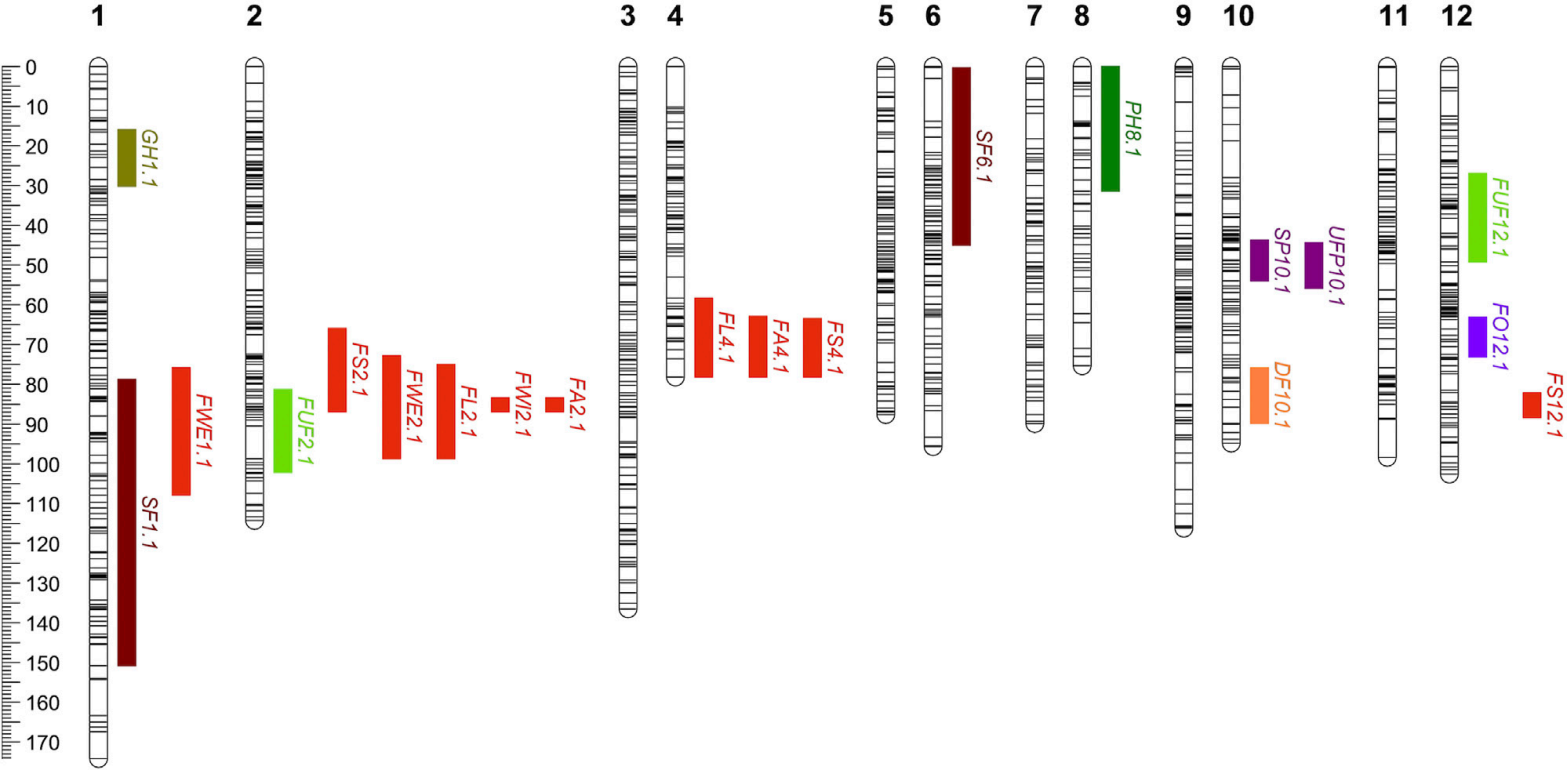
Distribution of the QTLs identified in a 1023-SNP chili pepper genetic map (n = 153, F_2:3_ Puya × Chiltepin). The colored bars show the location of the QTLs (bar length corresponds to the QTL 1.8 support intervals). SP, stem pigmentation; PH, plant height; UFP, unripe fruit pigmentation; FO, fruit orientation; FUF, form of unripe fruit; SF, seedless fruit; DF, deciduous fruit; FWE, fruit weight; FL, fruit length; FWI, fruit width; FA, fruit area; FS, fruit shape; GH, growth habit.

The power of the mapping analysis allowed the detection of the most relevant components which consisted of a reduced number of loci that explained a large portion of the phenotypic variation. Thus, only 20 QTLs were identified for 13 traits (Table 4; Figure 4). For example, for some traits with a single QTL such as *steam pigmentation*, *fruit orientation* and *deciduous fruit*, the phenotypic variation explained was up to 50.72, 49.15% and 44.48% respectively, while for some traits with more than one QTL such as *fruit area* and *fruit shape* the total explained phenotypic variation was up to 44.39% and 41.6% respectively. The 20 QTLs were distributed among seven of the 12 chromosomes. Three regions on chromosomes 2, 4, and 10 harbored QTLs for multiple traits, which may be due to a common genetic basis (or linkage of several loci. QTLs for traits related to *fruit size and shape* (*FA2.1*, *FWI2.1*, *FL2.1*, *FWE2.1*, *FS2.1*, *FL4.1*, *FA4.1* and *FS4.1*) were co-localized in a region around 80 and 70 cM in chromosomes 2 and 4 respectively. Conversely, QTLs for *organ pigmentation* (*SP10.1* and *UFP10.1*) co-located in a region close to 50 cM in chromosome 10.

**TABLE 4:** Summary of the chili pepper QTLs identified for agronomic and domestication-related traits in a F_2_:_3_ Puya × Chiltepin mapping population (n-153). SP, stem pigmentation; PH, plant height; UFP, unripe fruit pigmentation, FO, fruit orientation; FUF, form of unripe fruit; SF, seedless fruit; DF, deciduous fruit; FWE, fruit weight; FL, fruit length; FWI, fruit width; FA, fruit area; FS, fruit shape; GH, growth habit.

Trait	Model summary			Individual QTL							1.8-LOD interval		
	Model	LOD	%Var	QTL ID	Chr	Pos (cM)	LOD	%Var*	A.E.**	D***	cM	Mb	Length (Mb)
SP	y ∼ Q1	16.59	50.72	*SP10.1*	10	48.68	16.59	50.72	4.23	2.53	43.71–53.98	113.9–187.05	73.05
PH	y ∼ Q1	4.61	17.85	*PH8.1*	8	0	4.61	17.85	−8.40	5.45	0–31.36	0.86–127.90	127.03
UFP	y ∼ Q1	8.26	31.64	*UFP10.1*	10	48.68	8.26	31.64	1.22	0.14	44.37–55.94	154.14–197.41	43.27
FO	y ∼ Q1	15.27	49.15	*FO12.1*	12	72.5	15.27	49.15	6.84	−4.42	63.08–73.15	192.3–204.04	11.73
FUF	y ∼ Q1+Q2	8.86	32.72	*FUF2.1*	2	87.6	4.28	14.22	1.30	−1.38	81.31–102.17	154.4–165.26	10.8
				*FUF12.1*	12	33.98	4.57	15.25	−1.35	−0.23	26.91–49.22	13.9–44.70	30.73
SF	y ∼ Q1 + Q2	9.52	26.58	*SF1.1*	1	109.72	5.52	14.42	−0.55	0.34	78.78–150.84	159.76–266.52	106.75
				*SF6.1*	6	22.36	4.24	10.82	−0.26	0.30	0.32–45.02	0.14–183.65	183.51
DF	y ∼ Q1	11.55	44.28	*DF10.1*	10	79.56	11.55	44.28	1.24	0.77	75.89–89.78	224.6–229.4	4.7
FWE	y ∼ Q1	5.27	22.98	*FWE2.1*	2	86.48	5.27	22.98	−2.66	−1.02	72.75–98.67	145.8–163.2	17.4
FL	y ∼ Q1 + Q2	7.2	37.93	*FL2.1*	2	82.75	7.2	26.36	−0.36	−0.19	75.04–98.67	149.5–163.2	13.6
				*FL4.1*	4	78.21	3.77	11.06	−0.17	−0.20	58.34–78.21	213.7–222.4	8.67
FWI	y ∼ Q1 + Q2	12.21	41.17	*FWI1.1*	1	103.18	4.19	11.76	−0.059	−0.028	75.77–107.89	156.5–219.9	63.3
				*FWI2.1*	2	86.48	8.98	28.10	−0.089	−0.070	83.41–86.94	155.7–157.7	2.03
FA	y ∼ Q1 + Q2	13.38	44.09	*FA2.1*	2	86.48	10.85	33.69	−0.42	−0.29	83.41–86.94	155.7–157.7	2.03
				*FA4.1*	4	71.31	4.03	10.70	−0.15	−0.26	62.87–78.21	215.7–222.4	6.72
FS	y ∼ Q1 + Q2 + Q3	13.35	44.01	*FS2.1*	2	79.68	4.81	13.04	−0.22	−0.028	65.87–86.94	140.5–157.7	17.18
				*FS4.1*	4	78.21	4.53	12.20	−0.13	−0.17	63.53–78.21	218–222.4	4.34
				*FS12.1*	12	84.814	5.85	16.22	−0.23	−0.081	82.12–88.36	217.1–2,253	8.25
GH	y ∼ Q1	7.58	27.40	*GH1.1*	1	21.25	7.58	27.40	−0.58	0.028	15.89–30.22	7.55–16	8.44

*%Var, Percentage of phenotypic variation explained; **A.E., Additive effect (if positive, effect is towards Chiltepin phenotype, otherwise towards Puya); ***Dominance.

The phenotypic variation explained by each QTL ranged from 10.70% (*FA4.1*) to 50.72% (*SP10.1*). All marker-trait associations found in this study explained at least 10% of the phenotyping variance; therefore, they can be considered as major QTLs (Han et al., 2016) and corresponds with the expectation that large-effect loci are important in domestication (Ross-Ibarra, 2005). In particular, QTLs for *stem pigmentation*, *orientation* and *fruit dehiscence* explained at least 40% of the phenotypic variation, this suggests the existence of a major gene of great effect that controls a large part of the phenotypic variation (also showed segregation patterns consistent with a single dominant gene). The same applied to *unripe fruit pigmentation*, although this QTL explained lower phenotypic variance (31.64%).

There was one major QTL for the *organ pigmentation* traits (*SP10.1* and *UFP10.1*), located at ∼50 cM on chromosome 10. For both *SP* and *UFP*, the QTL explained a high percentage of the phenotypic variation (>30%). In this case, the wild parent allele increased pigmentation in stem and fruit (Table 4; Supplementary Figures S2, S4), which agrees with the differences observed between both parents. For *plant height*, a single QTL (*PH8.1*) in chromosome 8 was identified, explaining 17% of the phenotypic variation, with the domesticated allele being associated with greater plant height. Similarly, a single major QTL, explaining 45% of the observed variation, was identified for *fruit orientation* in chromosome 12, with the wild allele associated with the erect fruit phenotype and the domesticated allele with the pendant fruit phenotype. Two QTLs were detected for *form of unripe fruit* on chromosomes 2 (*FUF2.1*) and 12 (*FUF12.1*), with the latter being the most significant. The QTL model (y ∼ QTL1 + QTL2) explained 32.72% of the phenotypic variation; individually they explained 14.22% and 15.25%, respectively. For *Seedless fruit*, two QTLs, *SF1.1* and *SF6.1*, were identified on chromosomes 1 and 6, respectively. The percentages of phenotypic variation explained by these QTLs were between 10.82% and 14.2%. For both *SF1.1* and *SF6.1*, the domesticated allele was associated with the seeded fruit phenotype). For *deciduous fruit*, a major QTL on chromosome 10 explained 44.28% of the phenotypic variation, with the *deciduous fruit* phenotype associated with the wild allele. Ten QTLs were identified for traits related to *fruit size and shape* (FWE, FL, FWI, FA and FS; in all cases, larger and elongated fruit were associated with the domesticated allele). Chromosome 2 harbored QTLs for all *fruit size* and *shape* traits*,* specifically*,* a region covering the interval 32.0–79.6 cM, which explained between 13.04% and 33.69% of the phenotypic variation for the different traits. A single QTL was identified for *growth habit* located on chromosome 1; this QTL explained 27.4% of the phenotypic variation, with the wild allele associated with perenniality.

Most of the QTL effects were consistent with the parental phenotypes. For 12 QTL associated with chili pepper domestication syndrome traits (*dehiscence*, *orientation*, *size and fruit shape*), the effect of the domesticated allele followed the domesticated parent phenotype. The same was observed for the three QTLs of *tissue pigmentation* and *growth habit* traits, which were highly contrasting between wild and domesticated peppers. This indicates that the wild allele produces a phenotype similar to the wild parent, and the domesticated allele produces a phenotype similar to the cultivated accession, as observed in most species (Ross-Ibarra, 2005). Although the direction of the two QTLs of *fruit sterility* is towards the domesticated genotype, it is difficult to establish a connection and it is likely that other more complex genetic mechanisms have greater relevance. For the *form of unripe fruit* phenotype, the wild and domesticated parents were expected to favor the smooth and wrinkled phenotypes, respectively. The QTL on chromosome 2 followed the parental pattern, while the sign of the effect of the QTL on chromosome 12 was contrary. Although the chiltepin parent can reach a great height in the wild, the mapping population was measured when plants with the domesticated genotype could have been taller due to their precocity. The direction of the only QTL detected for the *plant height* phenotype is towards the domesticated genotype.

In general, domesticated alleles were recessive wild alleles (Table 4; 14 of 20 QTLs showed this pattern). Notably, this pattern was prevalent in domestication traits; 11 QTLs (for *fruit dehiscence* and *fruit shape/size*) out of 12 showed this trend, only in the *fruit orientation* phenotype was the wild allele recessive. Remarkably, the QTLs and the different patterns shared between them were consistent for the phenotypes that were tested in temporal replicates or in F2 and F3 individuals (Table 4; Supplementary Table S2).

## 4 Discussion

Continued selection and use during the domestication of *Capsicum* have produced a broad spectrum of evolutionary changes. These changes have led to the divergence of cultivated chili peppers from wild accessions. In this study, a QTL mapping study using wild and domesticated chili pepper germplasm was conducted to map domestication syndrome phenotypes as well other traits of agronomic interest. Variation in some of the evaluated traits was derived from direct selection during the domestication process (e.g., *fruit size/shape*, *fruit orientation* and *deciduous fruit*), However, for some other traits, the observed variation may have been produced indirectly (e.g., *organ pigmentation*, *plant architecture* and *leaf size/shape* traits) (Ross-Ibarra et al., 2007; Kumar et al., 2018). In our mapping population the greatest variation was observed for traits of both categories such as plant architecture (*plant height* and *main stem length*), *fruit size and shape*, *organ pigmentation* and *fruit orientation*.

The phenotypic contrast between the wild and domesticated chili pepper parental lines used in the current study allowed the analysis of a greater variety of traits of agronomic interest than in previous reports (Naegele et al., 2014; Chunthawodtiporn et al., 2018), which emphasizes the importance of using wild germplasm in mapping studies. In total 19 qualitative and quantitative traits related to plant architecture, fruit, and phenology were evaluated. Many of the trait frequency distributions resembled the expected segregation patterns for qualitatively and quantitatively plant traits. In particular, the ratio observed for *fruit orientation* suggests that the erect fruit trait is inherited as a recessive gene as previously reported in chili peppers (Lee et al., 2008) and other crops (Sun et al., 2019). Similarly, segregation patterns for chili pepper *stem pigmentation* in our study suggests that this trait is controlled by a single dominant gene, which agrees with previous *Capsicum* reports for pigmentation in foliage, flowers and fruit (Borovsky et al., 2004). On the other hand, a distortion in the expected segregation ratio (3:1) was observed for some traits such as the *form of unripe fruit*, *unripe fruit pigmentation* and *growth habit*. This distortion is probably due to the lack of phenotypic data for almost a third of the population due to semi-sterility, the (non-random) loss of plants in the F_2_ population (Supplementary Table S1), or the bias of qualitative phenotyping. As in most domesticated species (Ross-Ibarra, 2005; Diaz-Valenzuela et al., 2020), the phenotypic data was skewed towards the wild phenotype in both qualitative and quantitative traits. Although this trend has been analyzed directly for target phenotypes during crop domestication, our results show that this trend is also present for non-target traits such as *branch angle*, *leaf traits*, *plant height*, *main stem length* and *stem pigmentation* (Figure 2B). These results suggest that the genetic changes produced by domestication affect non-target traits and that the inclusion of these traits is necessary for a better understanding of conscious and unconscious selection.

As expected, significant positive correlations were observed among traits related to *leaf*, *fruit shape/size* and *organ pigmentation*. Interestingly, negative correlations were observed between both *fruit shape and size* with traits associated with the wild phenotype (e.g., *fruit orientation* and *unripe fruit pigmentation*), which may be due to the relatively low rate of recombination in an F_2_ population. Notably, the possibility of analyzing traits such as *form of unripe fruit*, *seedless fruit*, *deciduous fruit* and *growth habit* which have not been analyzed in previous studies highlight the value of using exotic and wild accessions in mapping experiments.

To the best of our knowledge, this is the first genetic map derived from a cross between a wild pepper (*Capsicum annuum* var. *glabriusculum*) and domesticated pepper. This genetic map showed a great consistency (r > 0.99) with the cytological characteristics and physical architecture of the chili pepper genome (Kim et al., 2014). Moreover, a previously reported reciprocal translocation between chromosome 1 and 8 (Wu et al., 2009; Park et al., 2014) was confirmed. This major translocation in the domesticated genome differentiates it from its wild ancestor and other related species such as *C. frutescens* and *C. chinense* (Wu et al., 2009). It has been suggested that, as in other species (Jáuregui et al., 2001; Farré et al., 2011; Stathos and Fishman, 2014).), this translocation acts as a genetic barrier due to sterility, particularly between domesticated and ancestral forms (Dempewolf et al., 2012; Lee et al., 2016).

In chili pepper, variations in shape and increase in fruit size are the main changes observed in domesticated fruits, which is associated with the change in fruit orientation (from erect fruit in the ancestor to pendant fruit in domesticated forms). In this study, 10 QTLs were identified for *fruit size and shape* related traits, of which five were located in a common region of chromosome 2 (140.5–163.2 Mb). Several QTLs of *fruit size and shape* on chromosome 2 have been reported in different genetic backgrounds in chili peppers (Han et al., 2016; Chunthawodtiporn et al., 2018). The QTL on chromosome 2 co-localizes with the *ovate* gene which has been directly associated with *fruit shape and size* in pepper (Tsaballa et al., 2011). On the other hand, Individual QTLs for fruit-related traits have been previously reported on chromosome 4 (Lee et al., 2020), however, no cluster of QTLs has been indicated in this region as in this study. Similarly, QTL *FWE1.1* on chromosome 1 colocalizes with *FW-1* previously reported by Han et al., 2016, while there were no equivalent QTLs identified in chromosome 12 (*FS12.1*) with those previously reported for the corresponding traits. This may be explained by the differences in parentals, ours including a wild relative. A major QTL (*FO12.1*) was identified for the *fruit orientation* trait on chromosome 12, which agrees with previously reported associations (Lee et al., 2008; Han et al., 2016).

The adaptation of domesticated plants to agricultural environments led to the loss of useful alleles for wilder and more severe environments (Barchenger et al., 2019). In particular, organ pigmentation present in wild plants represents a useful protective mechanism against abiotic factors such as radiation (Winkel-Shirley, 2001). *Fruit and stem pigmentation* were analyzed in this population, and we detected a colocalized QTL on chromosome 10. Only unripe fruit pigmentation has been analyzed in previous studies (Han et al., 2016), in which the *A* gene has been identified as responsible for this phenotype (Borovsky et al., 2004); therefore, it is likely that both *unripe fruit pigmentation* and *stem pigmentation* share the same genetic basis. On the other hand, the perennial growth habit that characterizes wild plants changed to an annual growth habit in cultivated forms, which represents an advantage for their cultivation rather than for their survival in their natural environment. The phenotypic variation for the *growth habit* trait (GH) allowed the detection of a novel QTL (*GH1.1*) on chromosome 1. The loss of dispersal mechanisms was an important change that allowed the larger fruits to remain on the plant until they were harvested manually. A novel QTL linked to the *deciduous fruit* was identified in chromosome 10.

As mentioned previously, a translocation was detected between the wild and domesticated genomes, and to which can be attributed the sterility of some of the progeny observed in the population as in other species (Jáuregui et al., 2001). Two novel QTLs (*SF1.1* and *SF6.1*) for *sterility* were identified, which might be useful in breeding programs as well as for future evolutionary studies.

The results of the QTL analysis presented herein show that the genetics of *Capsicum* domestication follows the predominant patterns in most species; few loci of relatively large effect, preponderance of recessive domesticated alleles, clustering of QTLs, pleiotropic loci and effect of QTLs biased toward the domesticated phenotype for strongly selected traits. The number and effect of QTLs identified in this study suggests that *Capsicum* crop evolution was driven primarily by a few loci of large effect that may have caused major phenotypic leaps, as has been suggested in other species (Doebley and Stec, 1993; Koinange et al., 1996; Xiong et al., 1999). This condition of the phenotypes of interest could have made the selection more efficient and ultimately allowed a faster transformation towards the domesticated forms (Barton and Keightley, 2002). Because random mutations are likely to produce primarily loss of function, most domesticated traits have been reported as recessive traits that usually decrease fitness in the wild (Ladizinsky, 1985; Pitrat, 1986; Sogaard and von Wettstein-Knowles, 1987; Lester, 1989). Our results show that for traits directly related to domestication this pattern is true with few exceptions, 11 of 12 QTLs were recessive to the domesticated phenotype. Furthermore, for most polygenic traits, the gene action of the QTLs corresponded to a skew distribution towards the wild type (towards the lowest value) (Figure 2B). QTL clustering, a popular attribute in the genetics of species domestication, was also detected in this study. This pattern has been mostly interpreted as closely linked loci or as a pleiotropic for several traits. It has been proposed that this condition has been opportune during domestication since the QTL clusters would become fixed faster (Poncet et al., 1998). Among the traits involved in the domestication of *Capsicum*, *fruit shape and size* traits were the main objective. For all QTLs identified for these traits the effects are biased towards the domesticated phenotype presumably due to the strong directional selection exerted on these traits.

Our phenotypic and QTL information, specifically for fruit traits, follow the same patterns reported by Diaz-Valenzuela et al., 2020 at the transcriptome level for the F_1_ hybrid of our same population. The general observation of recessiveness biased towards the domesticated phenotype in our study is consistent with the bias of the entire transcriptome towards the wild parent. In addition, the authors propose that the phenotypic divergence is mainly influenced by trans regulation, which complements the findings of the genetic architecture of the traits analyzed in this study. The relevance of trans-acting regulation variation in the phenotypic transformation of this species could also partially explain the pleiotropic effects of selection during domestication on both target and non-target traits. Thus, the findings of both studies carried out on the domestication of *Capsicum* provide relevant information to support the role of the omnigenic model in this process.

This study provides novel insights on the genetic control of both agronomic traits and traits of evolutionary interest in *Capsicum*. Our results demonstrate that the genetics of *Capsicum* domestication follows the predominant patterns in most species. The genetic map revealed a reciprocal translocation which is a genetic barrier mechanism that commonly accompanies domestication and contributes to differentiation between crops and their ancestors on short evolutionary scales. The analysis of target and non-target traits suggests that their consideration will allow a better understanding of crop evolution and will facilitate the design of strategies for the use of wild material in breeding processes. Additionally, the phenotypic variation observed in our population allowed the detection of QTLs for a wide variety of traits, including several that were explored for the first time (FUF, SF, DF and GH). Also, due to the role of structural variations in environmental adaptation (Huang et al., 2021; Songsomboon et al., 2021), the observed translocation between chromosome 1 and 8 may have been involved in the dispersal of *Capsicum* accessions to different agroecosystems. The confirmation of this hypothesis and experimental validation of genes identified within our QTLs will provide valuable information in the history of the less-known *Capsicum* domestication compared to other Mesoamerican crops.

## Data Availability

The original contributions presented in the study are included in the article/Supplementary Material, further inquiries can be directed to the corresponding authors.
