# Supplementary figures and images for: Genetic analysis and QTL mapping of domestication-related traits in chili pepper (Capsicum annuum L.)
Source: Front Genet. 2023 May 15;14:1101401. doi: 10.3389/fgene.2023.1101401 (PMC10225550; doi:10.3389/fgene.2023.1101401)

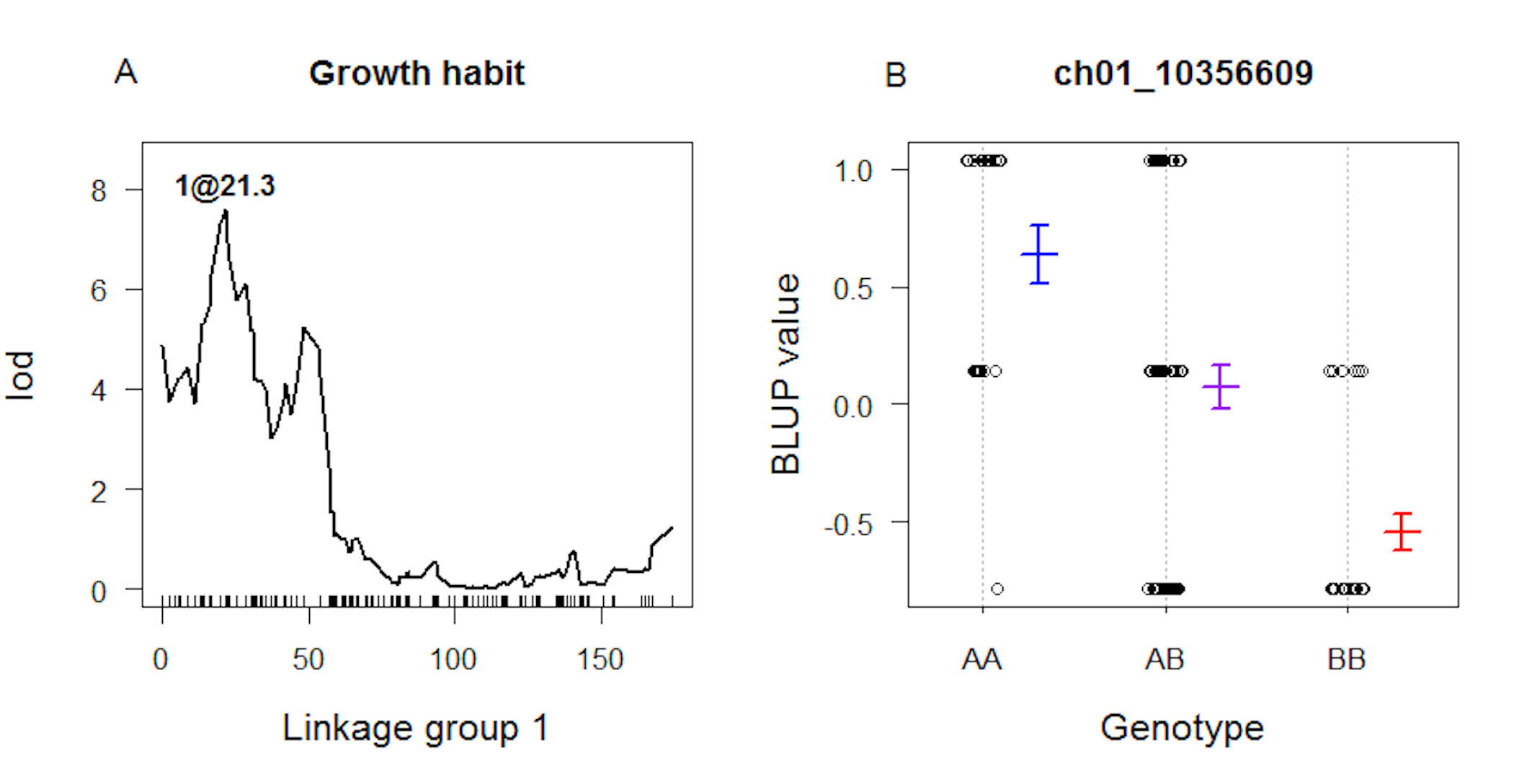

Supplement: Supplementary file 1 [file Image14.png]

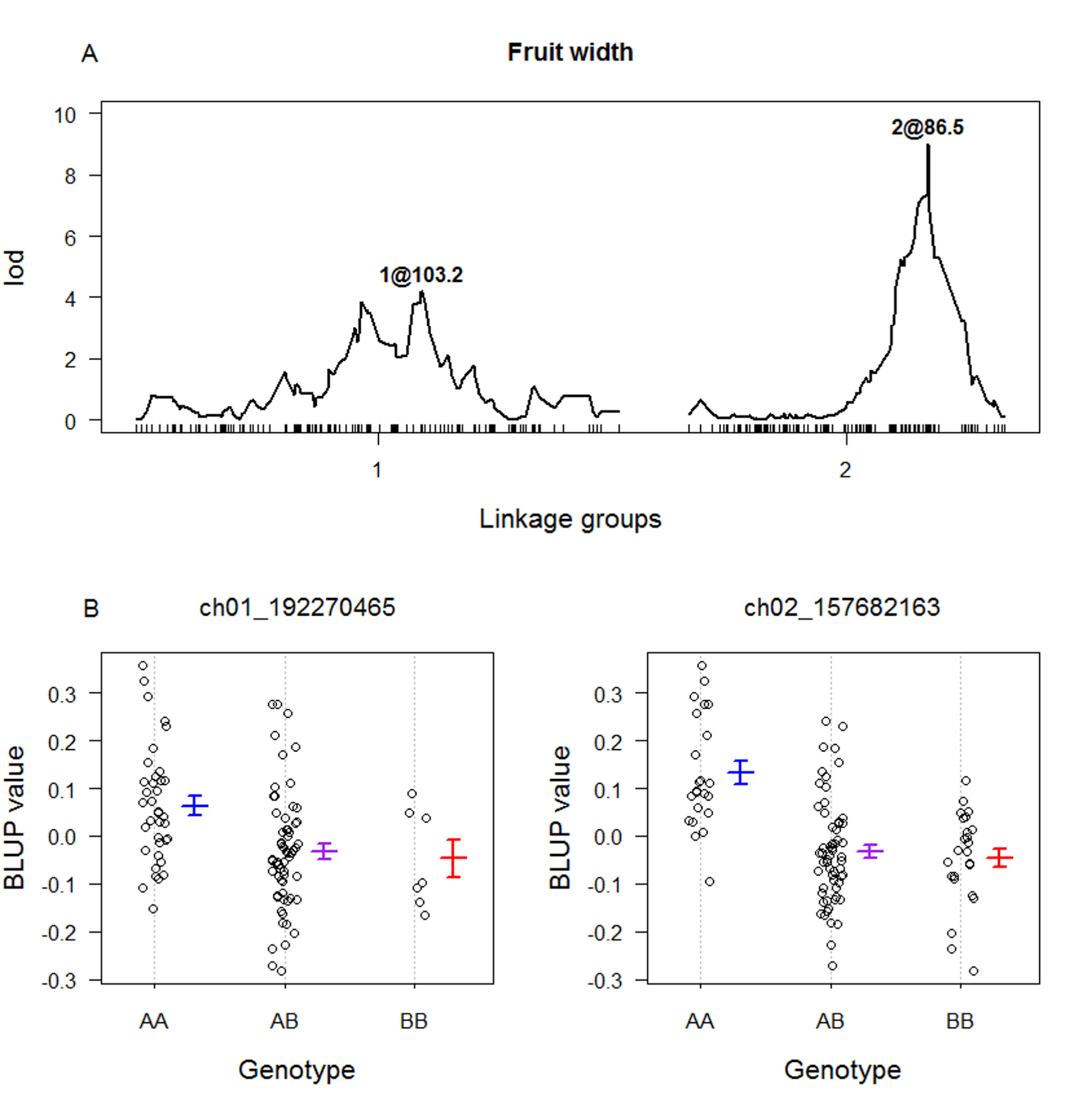

Supplement: Supplementary file 2 [file Image11.png]

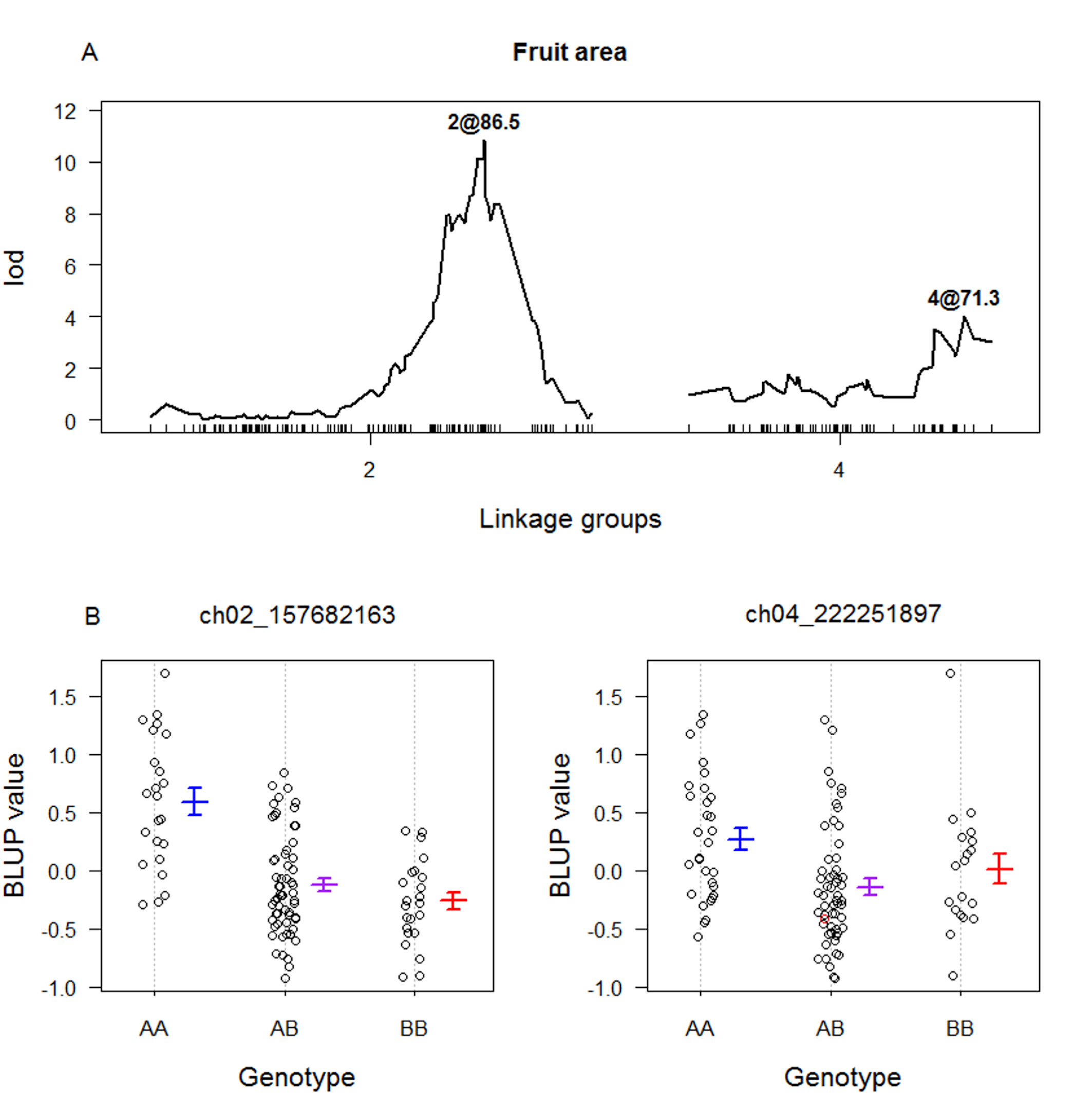

Supplement: Supplementary file 4 [file Image12.png]

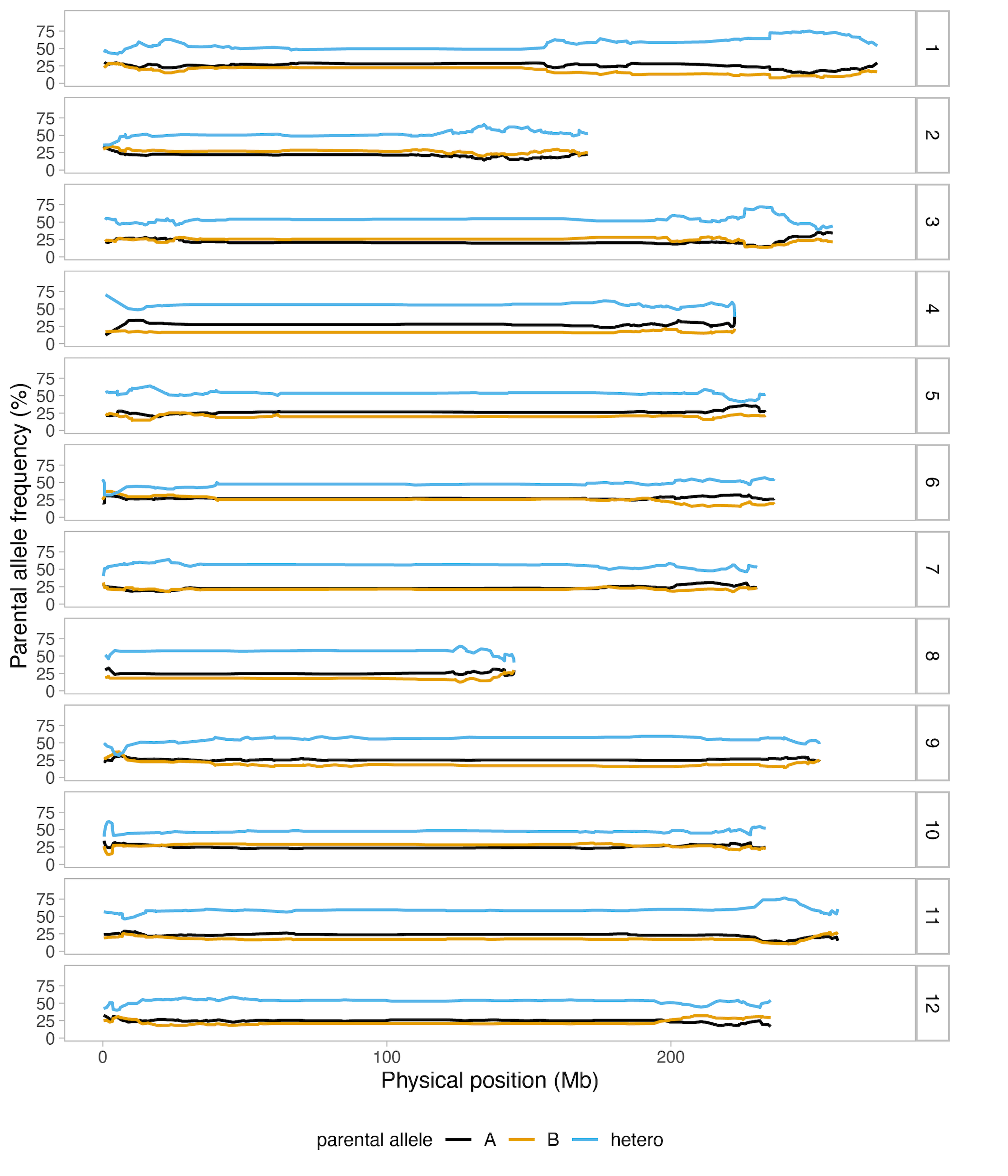

Supplement: Supplementary file 5 [file Image1.TIF]

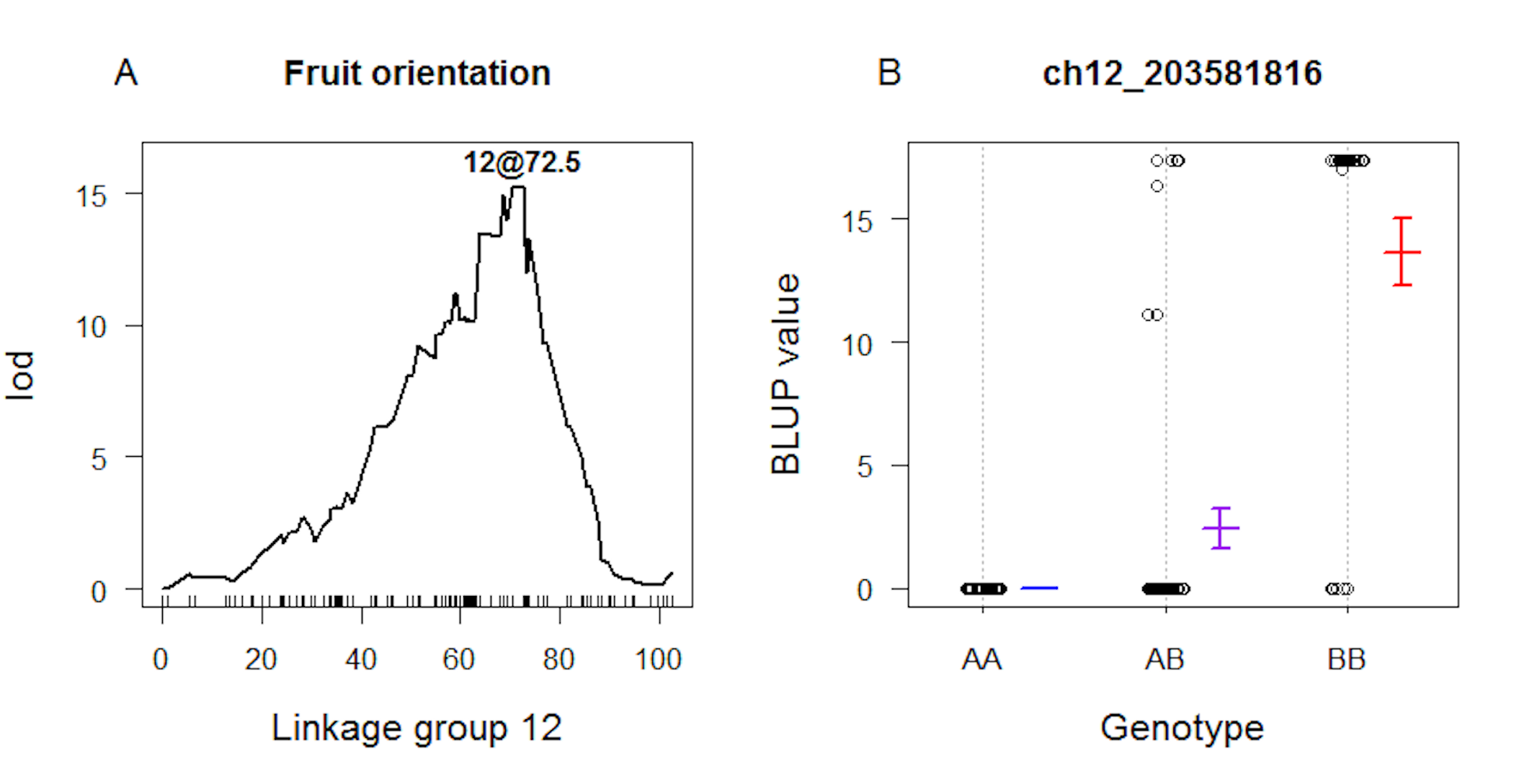

Supplement: Supplementary file 6 [file Image5.PNG]

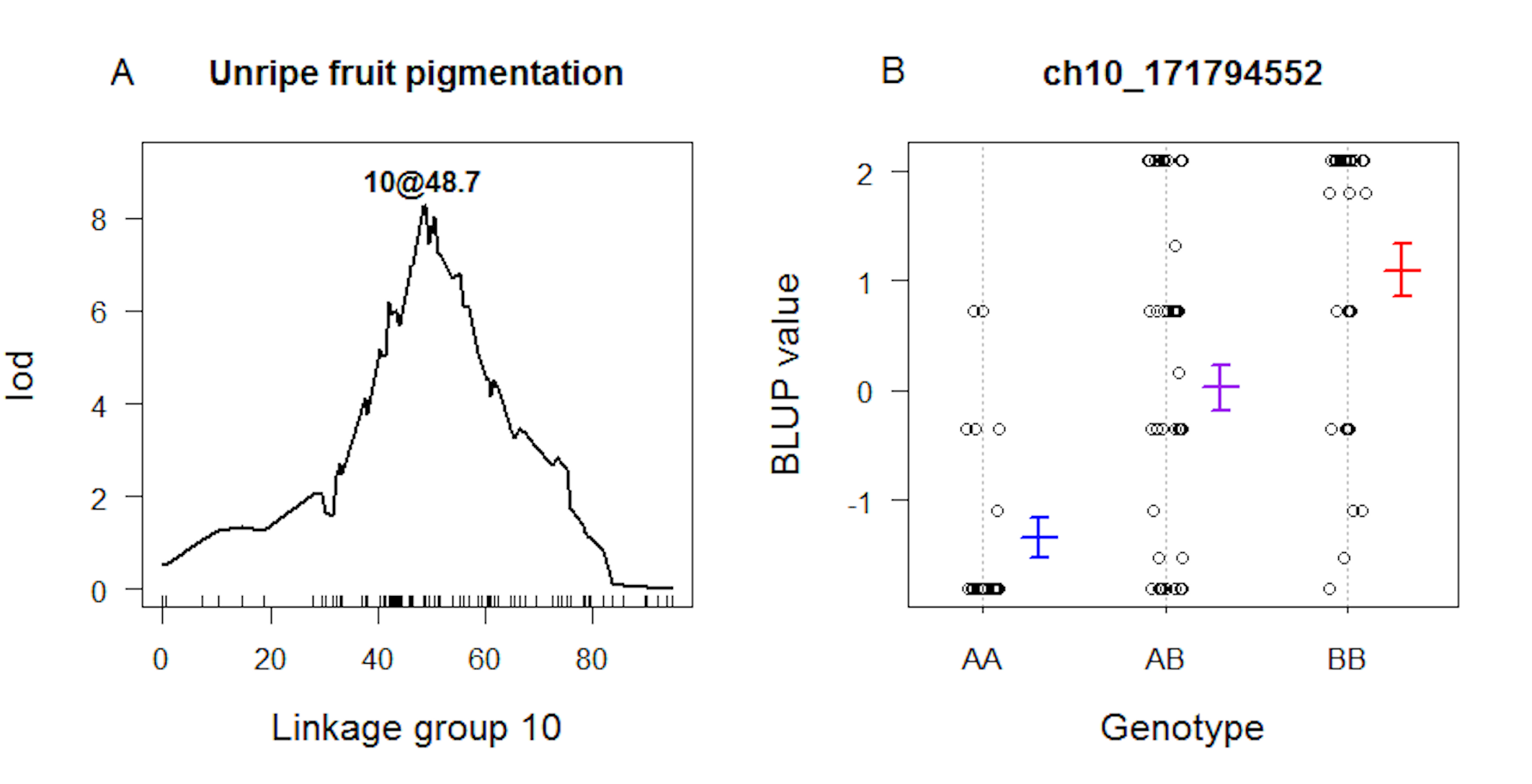

Supplement: Supplementary file 7 [file Image4.PNG]

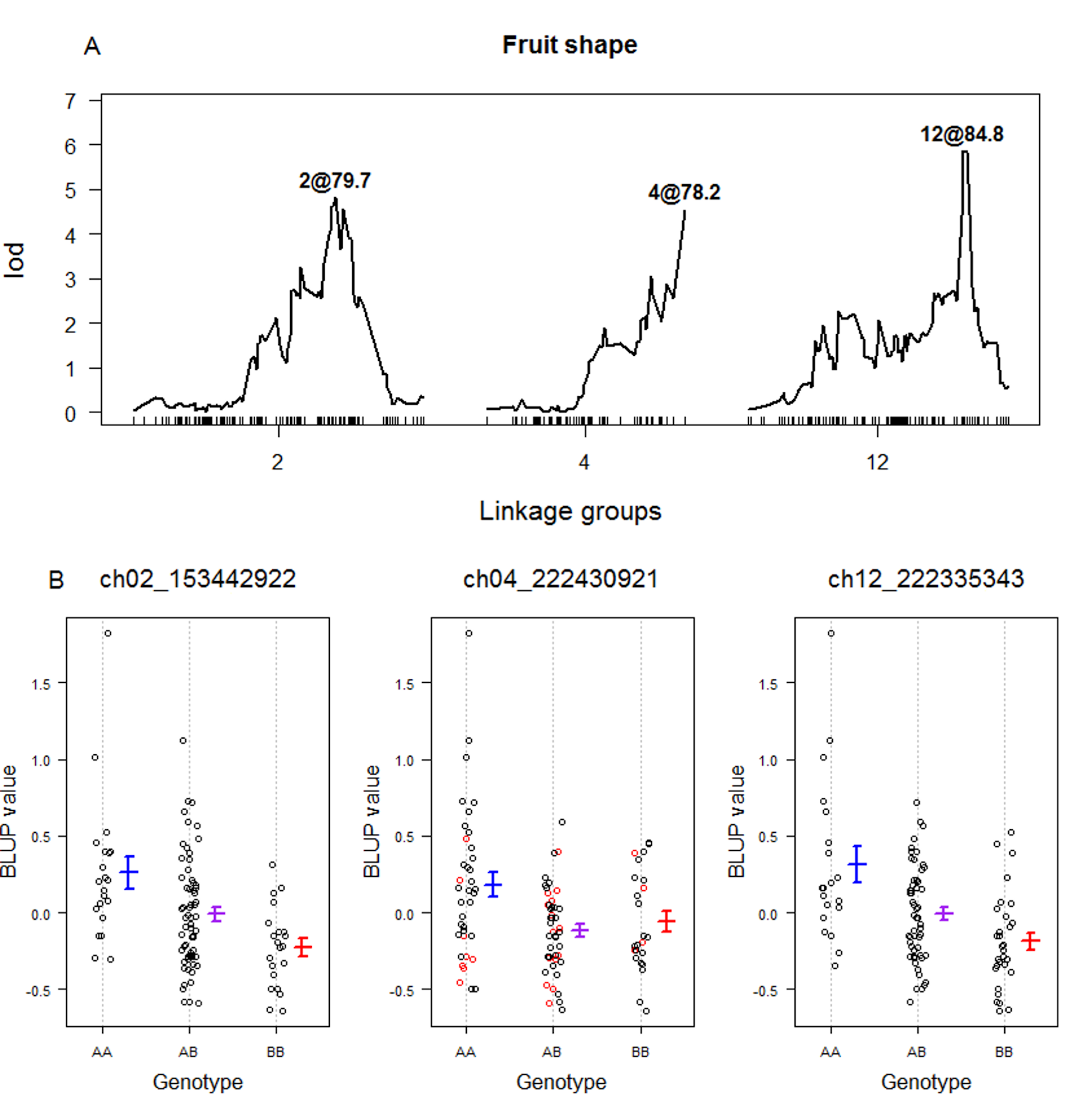

Supplement: Supplementary file 8 [file Image13.png]

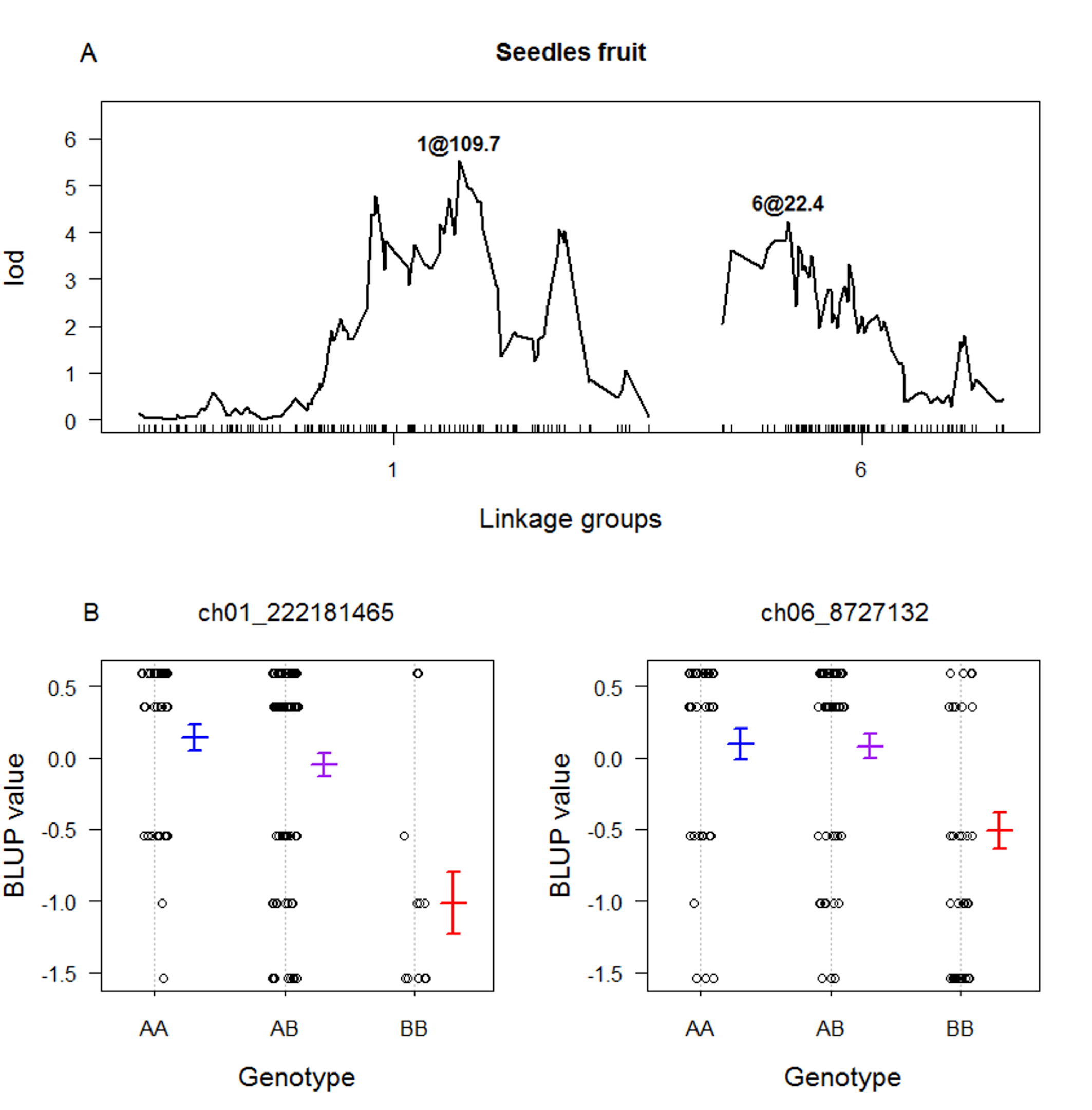

Supplement: Supplementary file 9 [file Image7.PNG]

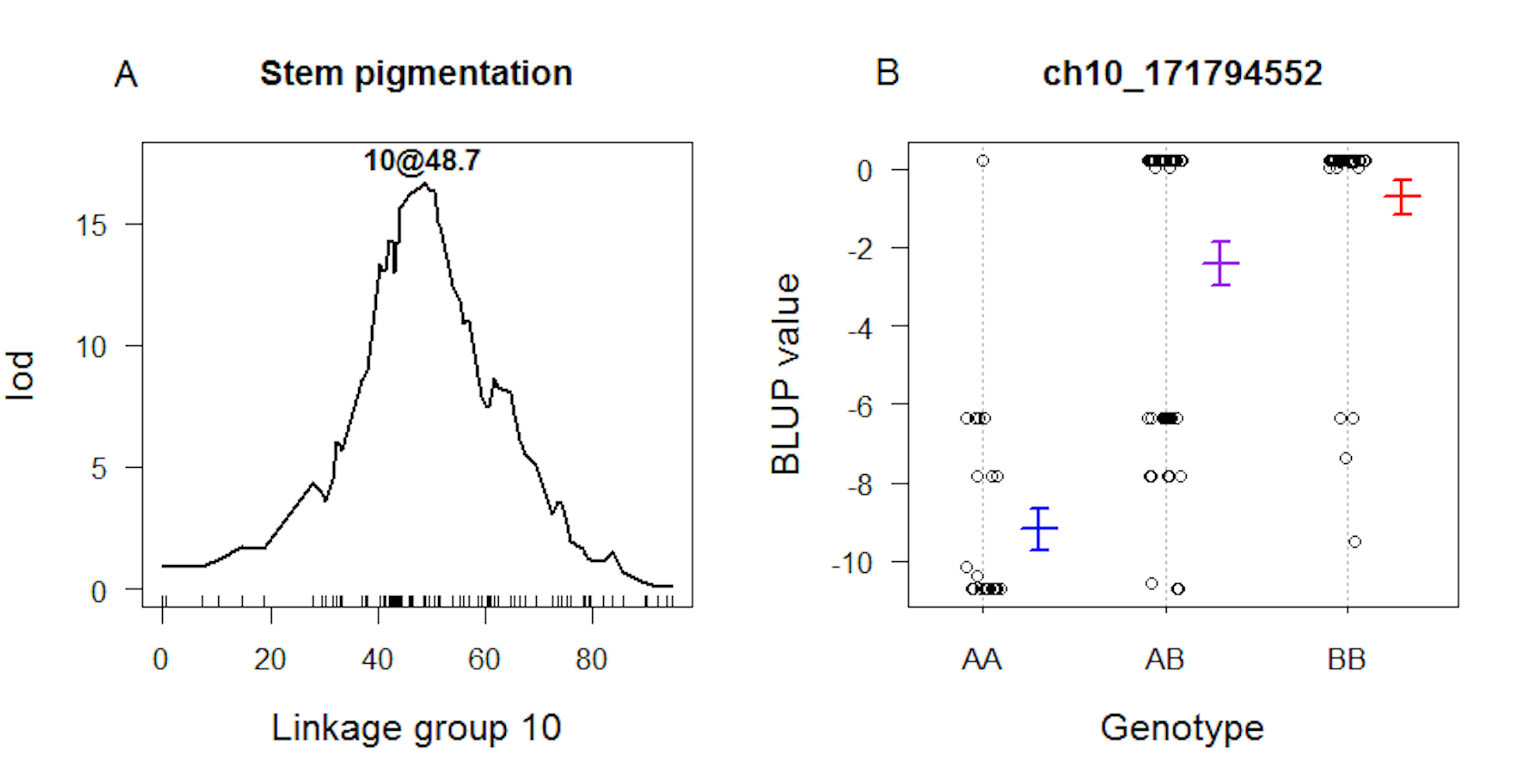

Supplement: Supplementary file 10 [file Image2.PNG]

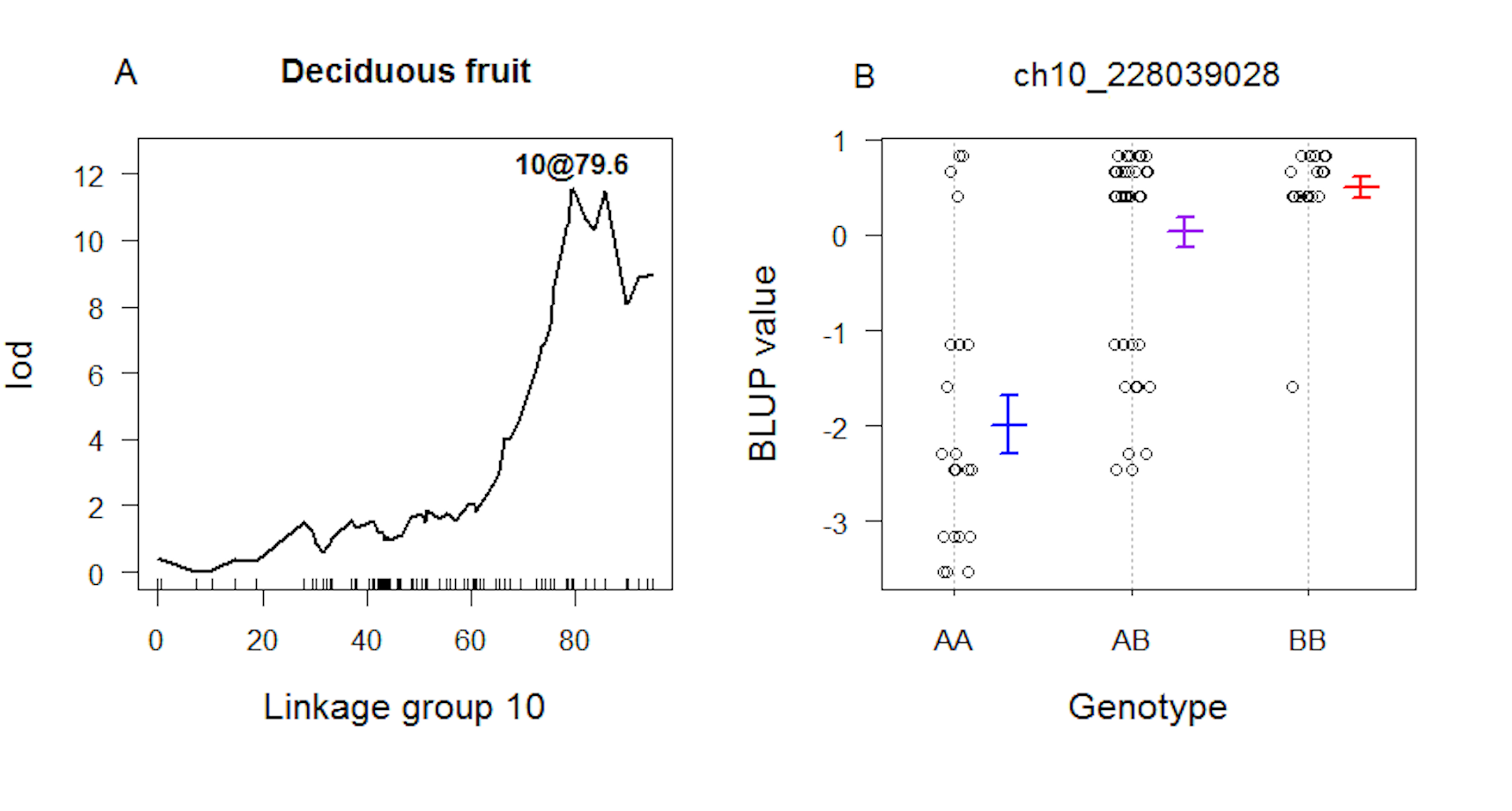

Supplement: Supplementary file 11 [file Image8.PNG]

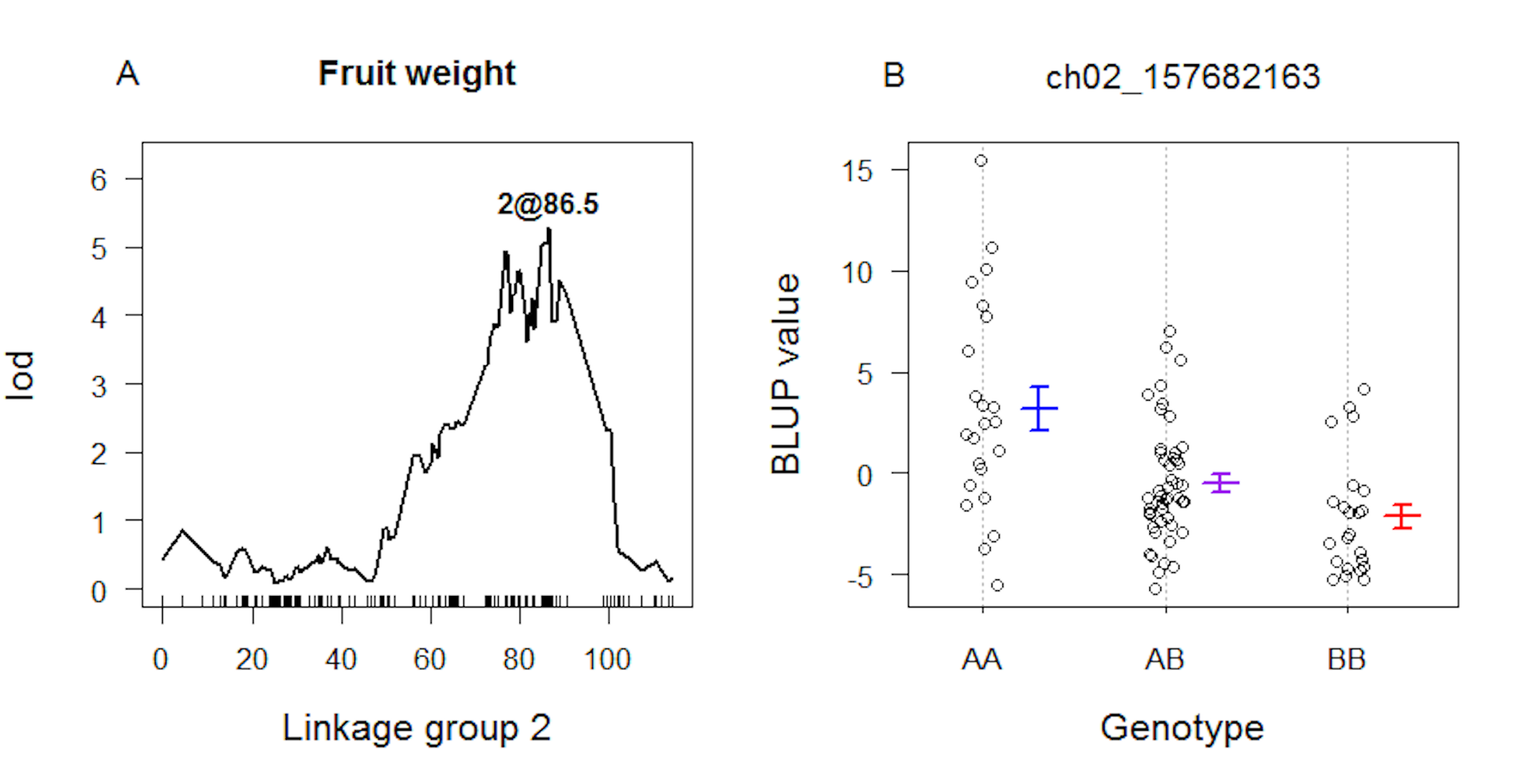

Supplement: Supplementary file 12 [file Image9.png]

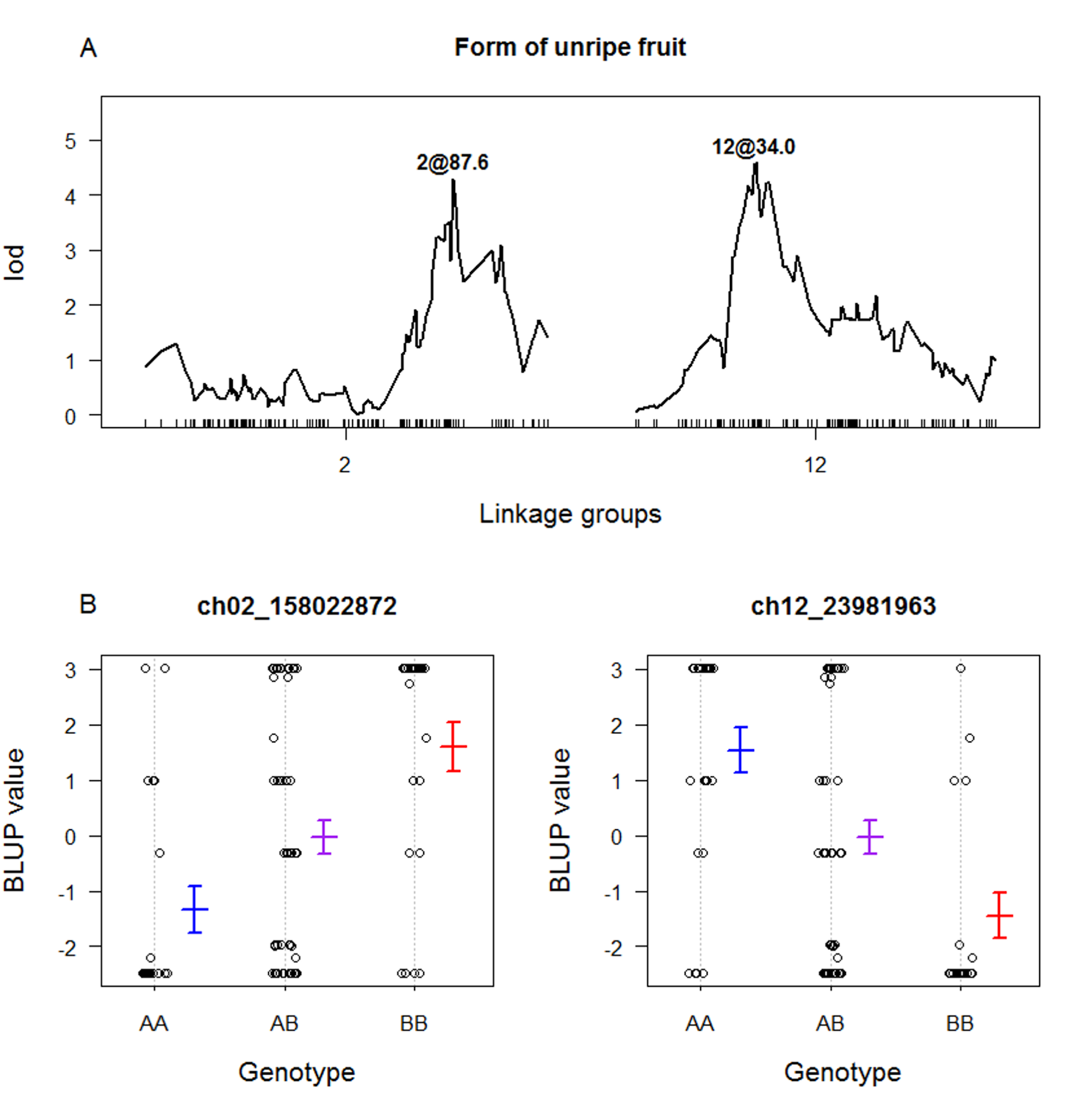

Supplement: Supplementary file 13 [file Image6.PNG]

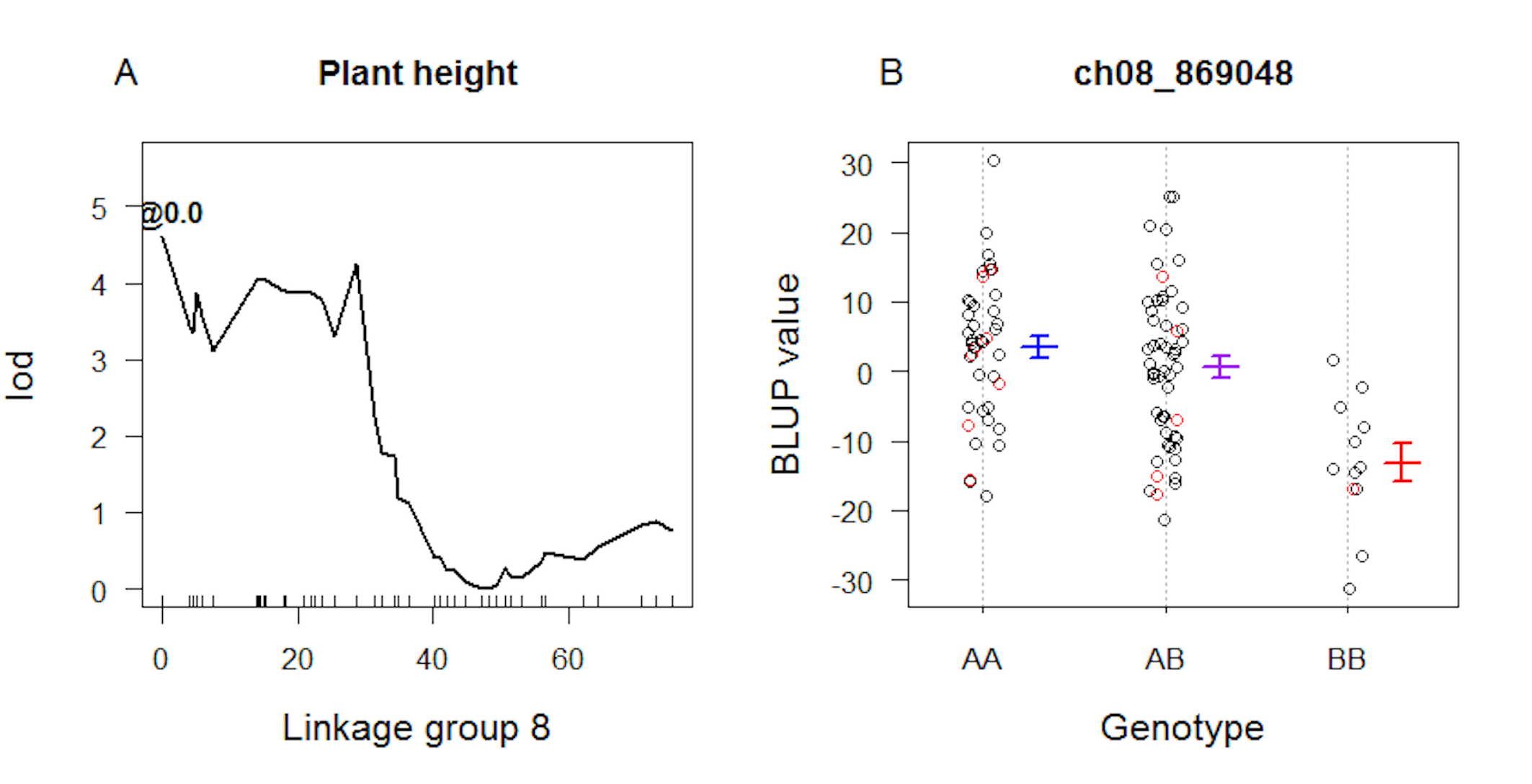

Supplement: Supplementary file 14 [file Image3.PNG]

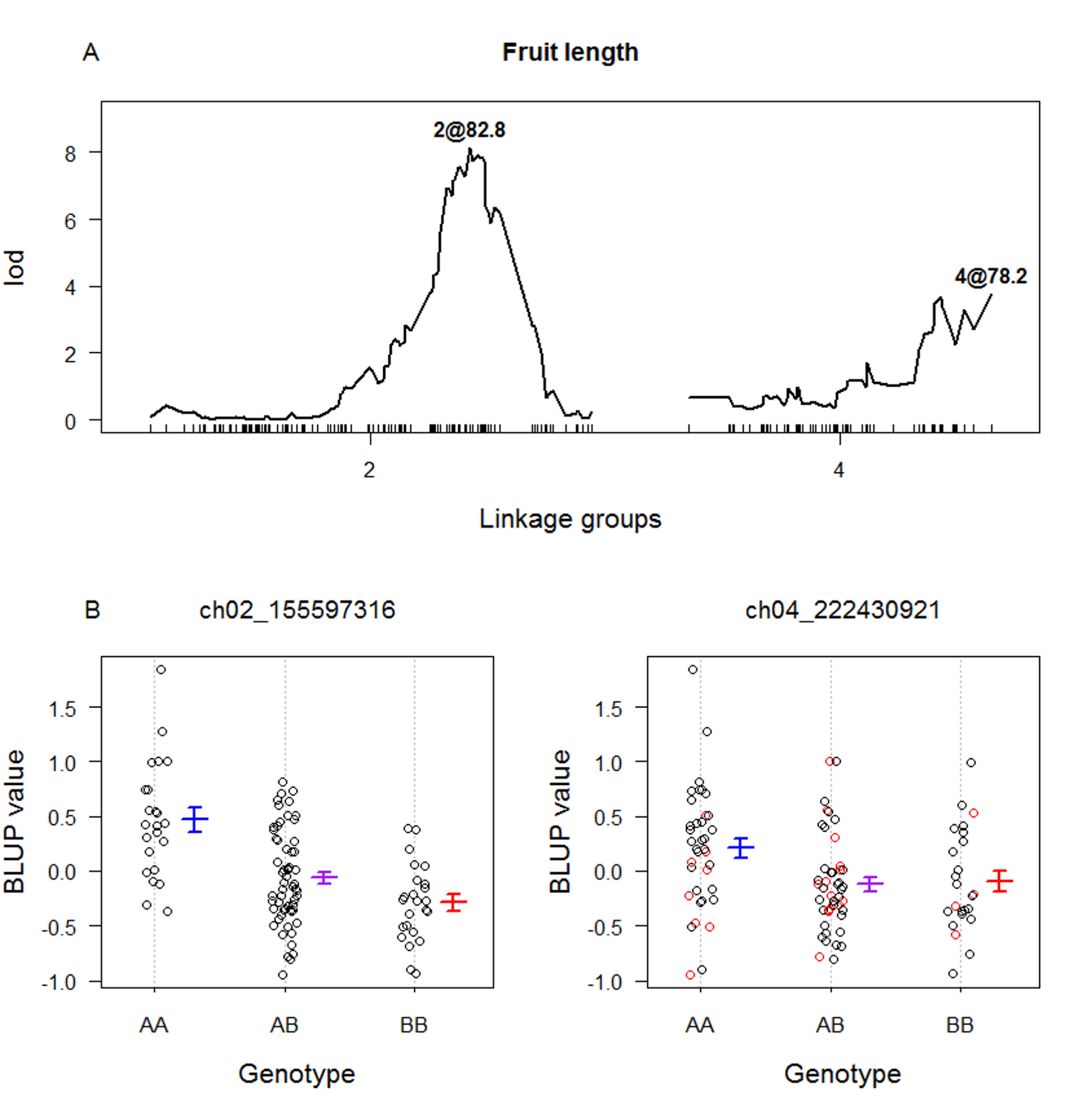

Supplement: Supplementary file 15 [file Image10.png]
