# Supplementary material for: Genetic analysis and QTL mapping of domestication-related traits in chili pepper (Capsicum annuum L.)
Source: Front Genet. 2023 May 15;14:1101401. doi: 10.3389/fgene.2023.1101401 (PMC10225550; doi:10.3389/fgene.2023.1101401)
Supplement: Supplementary file 3 [file Table1.docx]

**Supplementary Table 1.** Number of genotypic lines with phenotypic data available for each trait evaluated in a F_2_:_3_ Puya × Chiltepin mapping population (n=153).

| Trait | Genotypes with phenotypic data |
| --- | --- |
| Stem pigmentation | 108 |
| Plant height | 108 |
| Main stem length | 109 |
| Branch angle | 108 |
| Unripe fruit pigmentation | 100 |
| Fruit orientation | 104 |
| Form of unripe fruit | 103 |
| Seedless fruit | 142 |
| Deciduous fruit | 90 |
| Fruit weight | 93 |
| Fruit length | 107 |
| Fruit width | 107 |
| Fruit area | 107 |
| Fruit shape | 107 |
| Leaf length | 80 |
| Leaf width | 80 |
| Leaf area | 80 |
| Leaf shape | 80 |
| Growth habit | 109 |

**Supplementary Table 2**. Summary of the chili pepper QTLs identified for traits evaluated in the F_2_ Puya × Chiltepin mapping population (n=153). SP, stem pigmentation; UFP, unripe fruit pigmentation; FO, fruit orientation.

| **Trait** | **Model summary** | | | **Individual QTL** | | | | | | | **1.8-LOD interval** | | |
| --- | --- | --- | --- | --- | --- | --- | --- | --- | --- | --- | --- | --- | --- |
|  | **Model** | **LOD** | **%Var** | **QTL ID** | **Chr** | **Pos (cM)** | **LOD** | **%Var*** | **A.E.**** | **D***** | **cM** | **Mb** | **Length (Mb)** |
| SP | y ~ Q1 | 7.72 | 30.42 | SP10.1 | 10 | 49.66 | 7.72 | 30.42 | 0.43 | 0.27 | 46.33 – 59.21 | 169.94 – 203.58 | 33.63 |
| UFP | y ~ Q1 | 9.32 | 35.77 | UFP10.1 | 10 | 48.68 | 9.32 | 35.77 | 0.58 | 0.13 | 46.33-59.21 | 169.94 – 203.58 | 33.63 |
| FO | y ~ Q1 | 13.76 | 41.55 | FO12.1 | 12 | 70.47 | 13.76 | 41.55 | 0.56 | -0.50 | 66.09 – 73.15 | 195.96 – 204.04 | 8.07 |

*%Var, Percentage of phenotypic variation explained; **A.E., Additive effect (if positive, effect is towards Chiltepin phenotype, otherwise towards Puya); ***Dominance.
